# 5-ethyl-2’-deoxyuridine fragilizes *Klebsiella pneumoniae* outer wall and facilitates intracellular killing by phagocytic cells

**DOI:** 10.1371/journal.pone.0269093

**Published:** 2022-10-31

**Authors:** Estelle Ifrid, Hajer Ouertatani-Sakouhi, Tania Jauslin, Sebastien Kicka, Gianpaolo Chiriano, Christopher F. Harrison, Hubert Hilbi, Leonardo Scapozza, Thierry Soldati, Pierre Cosson

**Affiliations:** 1 Department of Cell Physiology and Metabolism, Faculty of Medicine, University of Geneva, Centre Médical Universitaire, Geneva, Switzerland; 2 Department of Biochemistry, Faculty of Science, University of Geneva, Geneva, Switzerland; 3 Pharmaceutical Biochemistry, School of Pharmaceutical Sciences, University of Geneva, Geneva, Switzerland; 4 Max von Pettenkofer Institute, Department of Medicine, Ludwig-Maximilians University Munich, Munich, Germany; 5 Institute of Medical Microbiology, Department of Medicine, University of Zürich, Zürich, Switzerland; Bryant University, UNITED STATES

## Abstract

*Klebsiella pneumoniae* is the causative agent of a variety of severe infections. Many *K*. *pneumoniae* strains are resistant to multiple antibiotics, and this situation creates a need for new antibacterial molecules. *K*. *pneumoniae* pathogenicity relies largely on its ability to escape phagocytosis and intracellular killing by phagocytic cells. Interfering with these escape mechanisms may allow to decrease bacterial virulence and to combat infections. In this study, we used *Dictyostelium discoideum* as a model phagocyte to screen a collection of 1,099 chemical compounds. *Phg1A* KO *D*. *discoideum* cells cannot feed upon *K*. *pneumoniae* bacteria, unless bacteria bear mutations decreasing their virulence. We identified 3 non-antibiotic compounds that restored growth of *phg1A* KO cells on *K*. *pneumoniae*, and we characterized the mode of action of one of them, 5-ethyl-2’-deoxyuridine (K2). K2-treated bacteria were more rapidly killed in *D*. *discoideum* phagosomes than non-treated bacteria. They were more sensitive to polymyxin and their outer membrane was more accessible to a hydrophobic fluorescent probe. These results suggest that K2 acts by rendering the membrane of *K*. *pneumoniae* accessible to antibacterial effectors. K2 was effective on three different *K*. *pneumoniae* strains, and acted at concentrations as low as 3 μM. K2 has previously been used to treat viral infections but its precise molecular mechanism of action in *K*. *pneumoniae* remains to be determined.

## Introduction

*Klebsiella pneumoniae* is a Gram-negative bacterium responsible for a variety of infections such as pneumonia and urinary tract infections. This opportunistic pathogen is frequently acquired in hospitals by patients with impaired immunity, but community-acquired infections are also common. The emergence of antibiotic-resistant strains represents a life-threatening risk for infected patients, and urgently requires the development of new anti-bacterial therapies [1]. However, development of new antibiotics with novel targets has proved arduous [2], and new screening strategies should be explored. Compounds that inhibit bacterial virulence could in principle be a potential alternative to classical antibiotics [3], although none have yet been fully developed.

Bacterial virulence is broadly defined as the ability of a specific bacterial strain to cause a disease in a given host. It reflects the equilibrium between the pathogenic potential of a bacterium and the host defense systems. A variety of model hosts can be used to assess the virulence of bacteria, ranging from mice to *Drosophila melanogaster* flies, *Caenorhabditis elegans* nematodes, *Tetrahymena pyriformis* ciliate protozoans and *Dictyostelium discoideum* amoebae [4–7]. For both practical and ethical reasons, non-mammalian hosts are more amenable to phenotypic screening, and have been used to identify new bacterial virulence genes [8] or inhibitors of bacterial virulence [9].

More specifically, *D*. *discoideum* has been used as a host to study the virulence of *Klebsiella pneumoniae* as well as the defense mechanisms of this model phagocytic cell [10–14]. In our laboratory we used a simple assay to monitor interaction between *D*. *discoideum* and *K*. *pneumoniae*: *D*. *discoideum* ingests non-pathogenic KpGE *K*. *pneumoniae* bacteria and can efficiently use them as a food source [15]. On the contrary, *phg1a* KO *D*. *discoideum* mutants kill ingested *K*. *pneumoniae* inefficiently. Consequently, when *K*. *pneumoniae* is the only food source available, *phg1a* KO *D*. *discoideum* grow very slowly [11]. This can be easily visualized by monitoring the ability of *D*. *discoideum* cells to grow and to form a phagocytic plaque devoid of live bacteria on a lawn of *K*. *pneumoniae*. This assay is robust and reproducible in middle-throughput format. It thus allows to identify *D*. *discoideum* mutants with poor antibacterial defense, *K*. *pneumoniae* mutants with decreased virulence, or chemical compounds affecting the host-pathogen interaction. Using this assay, the *kil2* KO *D*. *discoideum* strain was initially identified as a mutant exhibing poor growth in the presence of *K*. *pneumoniae*, and Kil2 was later shown to be essential for efficient intracellular killing of *K*. *pneumoniae* [16]. Similarly, *phg1A* KO cells grow more efficiently on a lawn of *K*. *pneumoniae* in which *waaQ* or *wbbM* are genetically inactivated, perturbing the synthesis of bacterial lipopolysaccharides (LPS) and reducing bacterial virulence [11]. Genetic inactivation of *waaQ* results in the production of LPS with an altered core [17]. When *wbbM* is genetically inactivated, the LPS is devoid of its O-antigen polysaccharide [18].

In this study, we used *phg1A* KO *D*. *discoideum* cells to identify chemical inhibitors of *K*. *pneumoniae* virulence. We identified and characterized 5-ethyl-2’-deoxyuridine (K2), as a compound facilitating intracellular killing of ingested *K*. *pneumoniae*. K2 did not exert an antibiotic activity, but it increased the permeability of the bacterial cell envelope and rendered the bacteria more susceptible to intracellular killing in phagosomes.

## Results

### Identification of putative inhibitors of *K*. *pneumoniae* virulence

**Fig 1:**
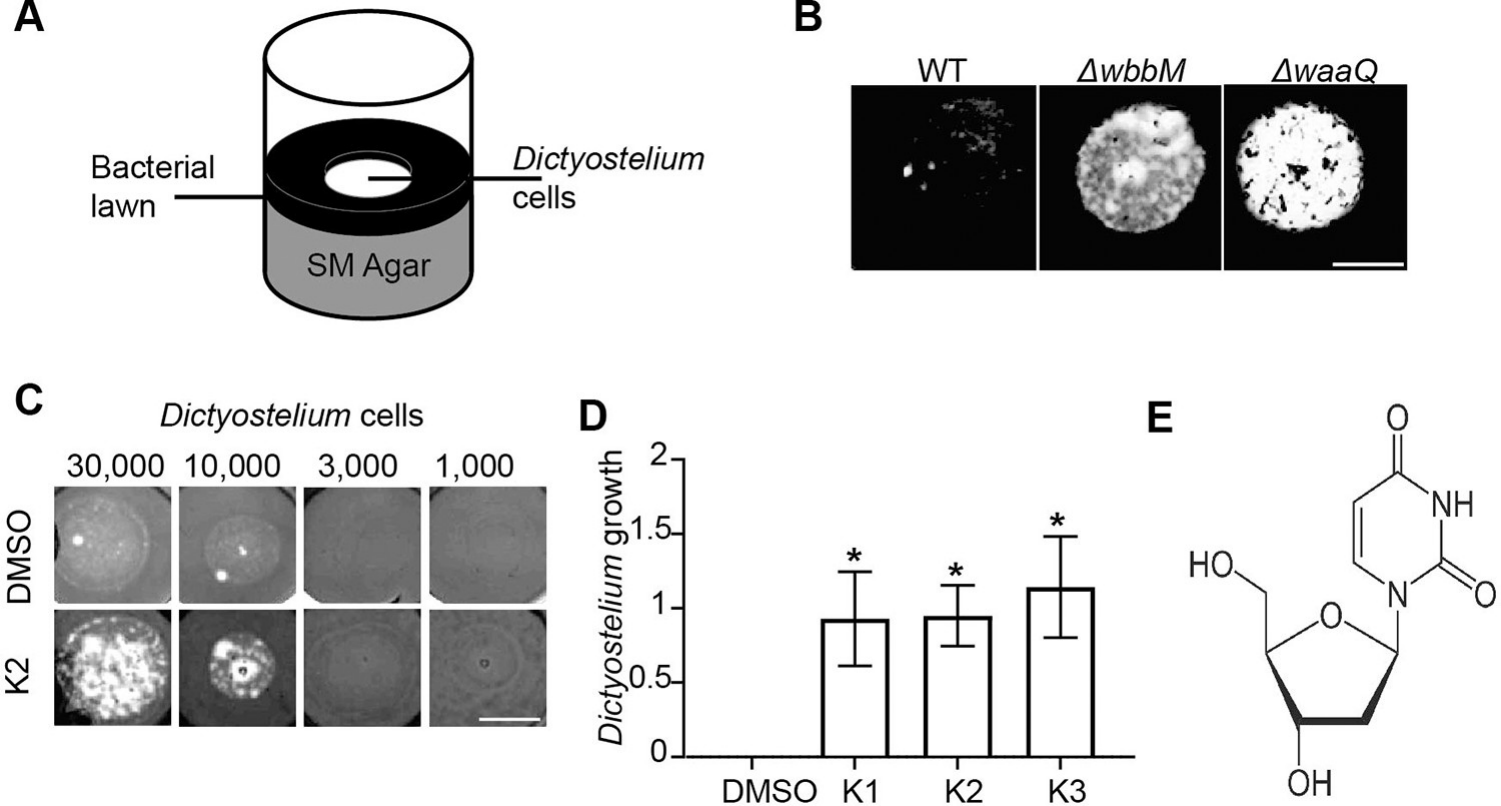
Three compounds affect the interaction between *K*. *pneumoniae* and phagocytic amoebae. **A.** *D*. *discoideum* cells were deposited on a lawn of *K*. *pneumoniae* and allowed to form a phagocytic plaque for 10 days. **B.** *Phg1A* KO cells failed to grow on WT bacteria, but they grew readily on *K*. *pneumoniae* mutants with decreased virulence (Δ*waaQ*, Δ*wbbM*) (scale bar: 4mm). **C.** K2 increased the ability of *phg1a* KO cells to create phagocytic plaques in comparison with the negative control (DMSO) (scale bar: 4mm). **D.** The effect of each compound was scored from 4 (visible growth of 1,000 cells) to 0 (no growth of 30,000 cells) and the score of the negative control (DMSO) substracted. In this scale, the result shown in Fig 1C would score as a 0 for DMSO, and 2 for K2. Repeated experiments showed a high variability, but a significant effect for all three selected compounds (mean ± SEM; *: p<0.05; Kruskal-Wallis test, Dunn’s test. DMSO, K2: N = 10; K1, K3: N = 7 independent experiments). The original uncontrasted pictures are shown in S1 Fig in S1 File. **E.** Chemical structure of the K2 compound.

In order to identify compounds that perturb the interaction between *K*. *pneumoniae* and host cells, we used a simple assay to visualize growth of phagocytic *D*. *discoideum* cells on a lawn of *K*. *pneumoniae*. *K*. *pneumoniae* bacteria were deposited on Standard Medium nutrient agar, then 10,000 *D*. *discoideum* cells were deposited in the center of the well, and allowed to grow for 10 days (Fig 1A). As previously described, wild-type (WT) *D*. *discoideum* created a phagocytic plaque in the bacterial lawn [11]. On the contrary, *phg1A* KO cells were virtually unable to grow on a lawn of WT *K*. *pneumoniae* (Fig 1B). Genetic inactivation of LPS synthesis genes (*waaQ* or *wbbM*) decreased the virulence of *K*. *pneumoniae* and concomitantly restored growth of *D*. *discoideum* cells (Fig 1B). This observation indicates that this assay can detect a decrease in the virulence of *K*. *pneumoniae* bacteria. The same assay was then repeated adding to each well a test compound at a final concentration of 30 μM, to screen a collection of 1,099 mostly FDA-approved compounds. On the first round of screening 14 compounds were selected, and after re-testing, 3 confirmed hits, named K1 to K3, were selected for further studies (S2 Fig in S1 File). The three compounds were reordered and their effect was confirmed.

We then retested each of the confirmed hits in a more quantitative manner, depositing on a lawn of *K*. *pneumoniae* an increasing number of *phg1A* KO *D*. *discoideum* cells, from 1,000 to 30,000, and the growth of *D*. *discoideum* was scored in each experiment (Fig 1C). A score of 0 indicates that no growth of *D*. *discoideum* was observed, suggesting that the *K*. *pneumoniae* bacteria are virulent. On the contrary, a score of 4 indicates that even 1,000 cells were sufficient to create a phagocytic plaque in the bacterial lawn, suggesting that the virulence of *K*. *pneumoniae* bacteria was strongly decresased. In repeated experiments, all three compounds increased reproducibly and comparably the ability of *D*. *discoideum* cells to grow in the presence of *K*. *pneumoniae* (Fig 1D). The chemical structure of the K2 compound, on which this study is focused, in shown (Fig 1E).

**Fig 2:**
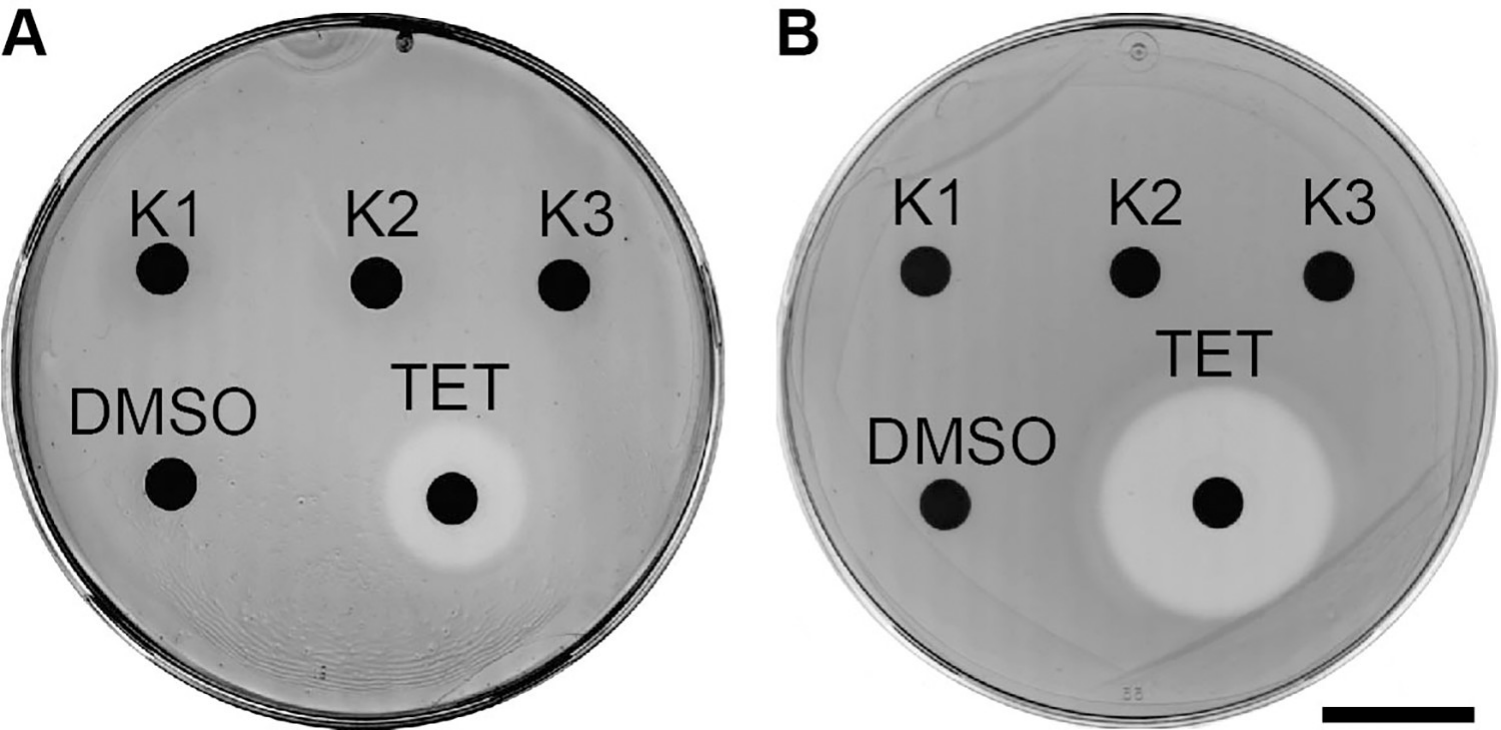
Selected compounds exhibit no antibiotic activity against *K*. *pneumoniae*. *K*. *pneumoniae* were plated on LB**- (A**) or SM- **(B)** agar plates. Paper discs with 20 μl of a 10 mM DMSO stock solution of each compound were then placed on the agar and the bacteria allowed to grow. After an overnight incubation at 25°C, a halo of bacterial growth inhibition was observed around a disk containing tetracycline (TET), but none of the selected compounds showed a similar effect (scale bar: 2 cm).

One trivial possibility would be that the selected compounds inhibit the growth of *K*. *pneumoniae*, and that this would account for the increased ability of *D*. *discoideum* cells to create a plaque in the thinner bacterial lawn. To assess this possibility, we tested directly the ability of each compound to inhibit growth of *K*. *pneumoniae*. For this, each compound was deposited on a disc at the surface of an LB-agar plate seeded with *K*. *pneumoniae* bacteria. Tetracycline was used as a positive control: it inhibited growth of *K*. *pneumoniae* as revealed by a halo of growth inhibition around the site of deposition (Fig 2). None of the selected compounds inhibited bacterial growth in this assay neither in LB (Fig 2A) nor in Standard Medium (Fig 2B).

### K2 facilitates the intracellular killing of *K*. *pneumoniae* by *D*. *discoideum* cells

**Fig 3:**
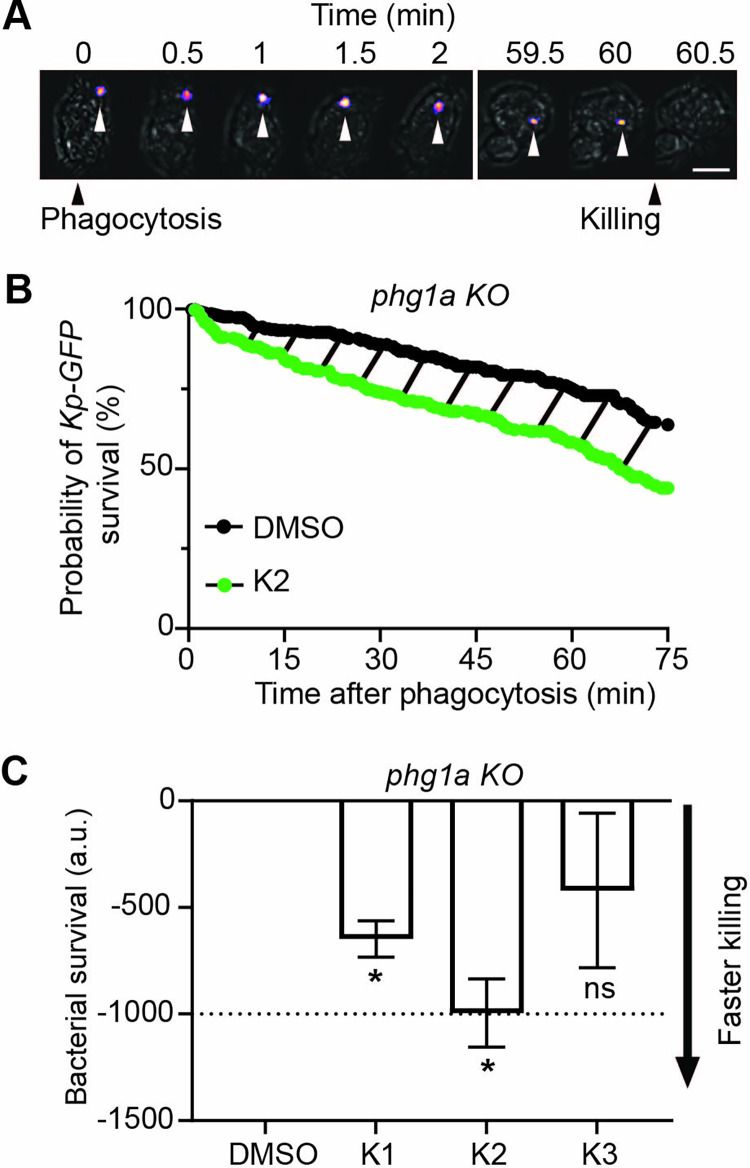
K2 increases the intracellular killing of *K*. *pneumoniae* by *phg1a KO* cells. **A.** Time-lapse images showing one representative example of a fluorescent *K*. *pneumoniae* ingested by a *D*. *discoideum phg1a* KO cell. The phase contrast and fluorescence pictures were superimposed, and the position of the fluorescent bacteria indicated with an arrowhead. Time 0 is defined as the time when the *D*. *discoideum* cell engulfs the bacteria (Phagocytosis). In this example, extinction of fluorescence was observed 60 minutes after ingestion (Killing) (scale bar: 6μm). Only the essential time points are shown, showing the moment when the bacteria was phagocytosed (t = 0), and when their fluorescence was lost (t = 60min). **B.** Survival of *K*. *pneumoniae* (%, Kaplan-Meier estimator) ingested by *phg1a* KO cells was decreased in the presence of the compound K2 (green) compared with the DMSO control (black). These two curves were obtained by combining the results of 6 independent experiments (total 180 bacteria for each condition). The dashed area represents the difference between the two survival curves. **C.** The Kaplan-Meier survival curves were determined in multiple independent experiments (30 bacteria for each condition) for the three tested compounds (30μM). The area under the curve (AUC) for each compound (K1-K3) and the control (DMSO) was determined for each experiment over 75 min, and the difference (corresponding to the dashed area in Fig 3B) was calculated. A figure inferior to zero indicates that the killing was faster in the presence of the compound than in the control (DMSO) condition. Two compounds, K1 and K2, increased significantly the intracellular killing of *K*. *pneumoniae* by *phg1a* KO cells (mean ± SEM; *: p<0.05; Kruskal-Wallis test, Dunn’s test. K1, K3: N = 3; DMSO; K2: N = 11 independent experiments).

The ability of *D*. *discoideum* cells to grow on a lawn of bacteria can be modulated in a number of ways, such as an change in phagocytic uptake, intracellular killing, or phagocyte motility. We first tested if selected compounds restored at least partially the ability of *phg1A* KO cells to kill ingested *K*. *pneumoniae*. For this, we measured intracellular killing of GFP-expressing *K*. *pneumoniae* by assessing fluorescence extinction following phagocytosis. Previous experiment have shown that the abrupt extinction of GFP fluorescence provides a good estimate of the moment at which ingested bacteria are killed [19]. Using this method, bacterial fluorescence is extinguished within a few minutes after phagocytosis in WT cells [19]. On the contrary, and in agreement with previous observations [11], ingested bacteria remained alive for a long time in *phg1A* KO cells: a typical example is shown, where bacterial killing occurred 60 min after ingestion in *phg1A* KO cells (Fig 3A). Multiple ingestion/killing events were recorded (30 per independent experiment), and the survival of bacteria following ingestion was determined: less than 50% of bacteria were killed within the first hour following their ingestion in *phg1A* KO cells (Fig 3B; N = 6 independent experiments, n = 180 ingested bacteria). When bacteria and cells were treated with K2, ingested bacteria were killed faster than in the absence of compound. To determine the statistical relevance of these observations, in each independent experiment, the area under the bacterial survival curve was determined, and the control value (DMSO) was substracted (dashed area in Fig 3B). A value below zero corresponds to a killing that is faster in the presence of the compound than in the control condition. The K1 and K2 compounds accelerated intracellular killing of *K*. *pneumoniae*, and the effect was strongest for K2 (Fig 3C). Consequently the rest of this study was focused on the K2 compound.

**Fig 4:**
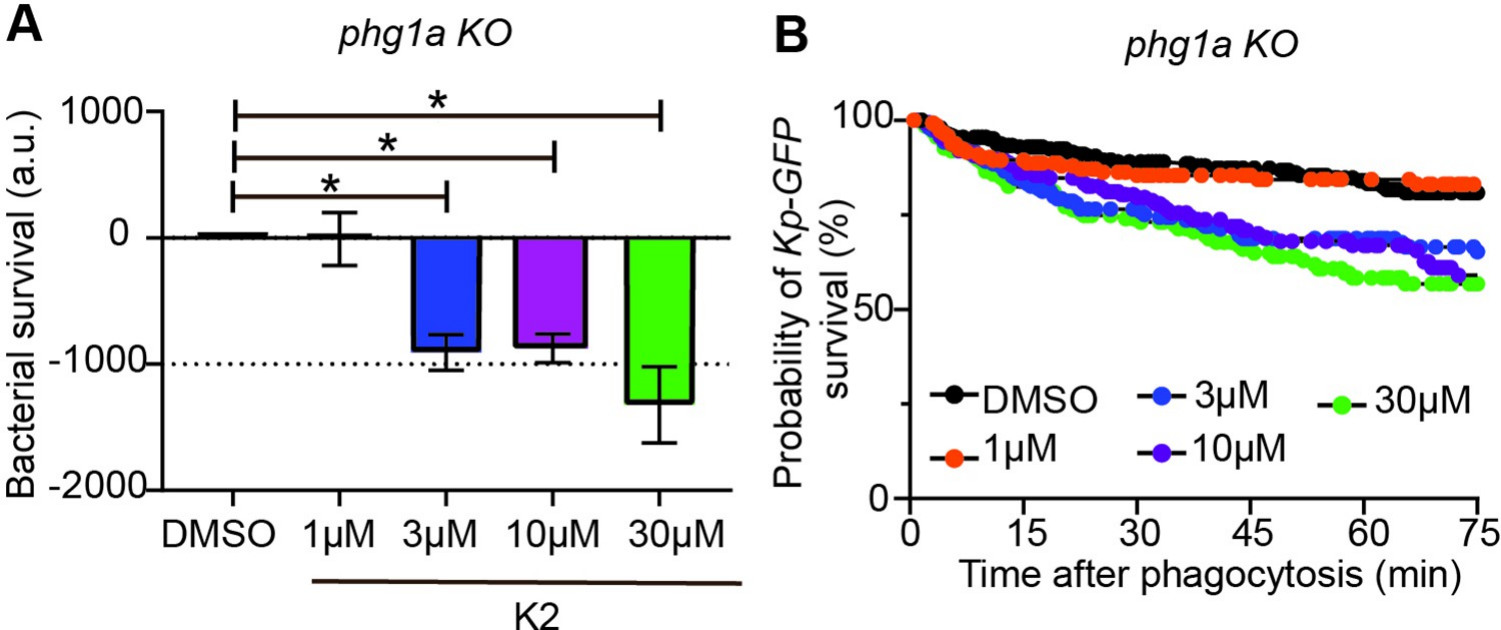
K2 is active at a concentration of 3 μM and above. **A.** The effect of K2 on the intracellular killing of *K*. *pneumoniae* was determined as described in the legend to Fig 3 at concentrations of K2 ranging from 1 to 30 μM. K2 increased intracellular killing of bacteria at 3 μM (blue), 10 μM (purple) and 30 μM (green) (mean ± SEM; *: p<0.05; Kruskal-Wallis test, Dunn’s test. DMSO, 3 μM and 10 μM: N = 6; 1 μM: N = 5; 30 μM: N = 4 independent experiments). **B.** The corresponding survival curves of ingested *K*. *pneumoniae* are shown.

In order to characterize the mode of action of K2, we next tested its effect on intracellular killing over a range of concentrations. K2 stimulated intracellular killing at concentrations of 30 μM, 10 μM and 3 μM, while no effect was observed at 1 μM (Fig 4A). This result was also apparent when the cumulative survival curves of ingested bacteria were compared (Fig 4B).

**Fig 5:**
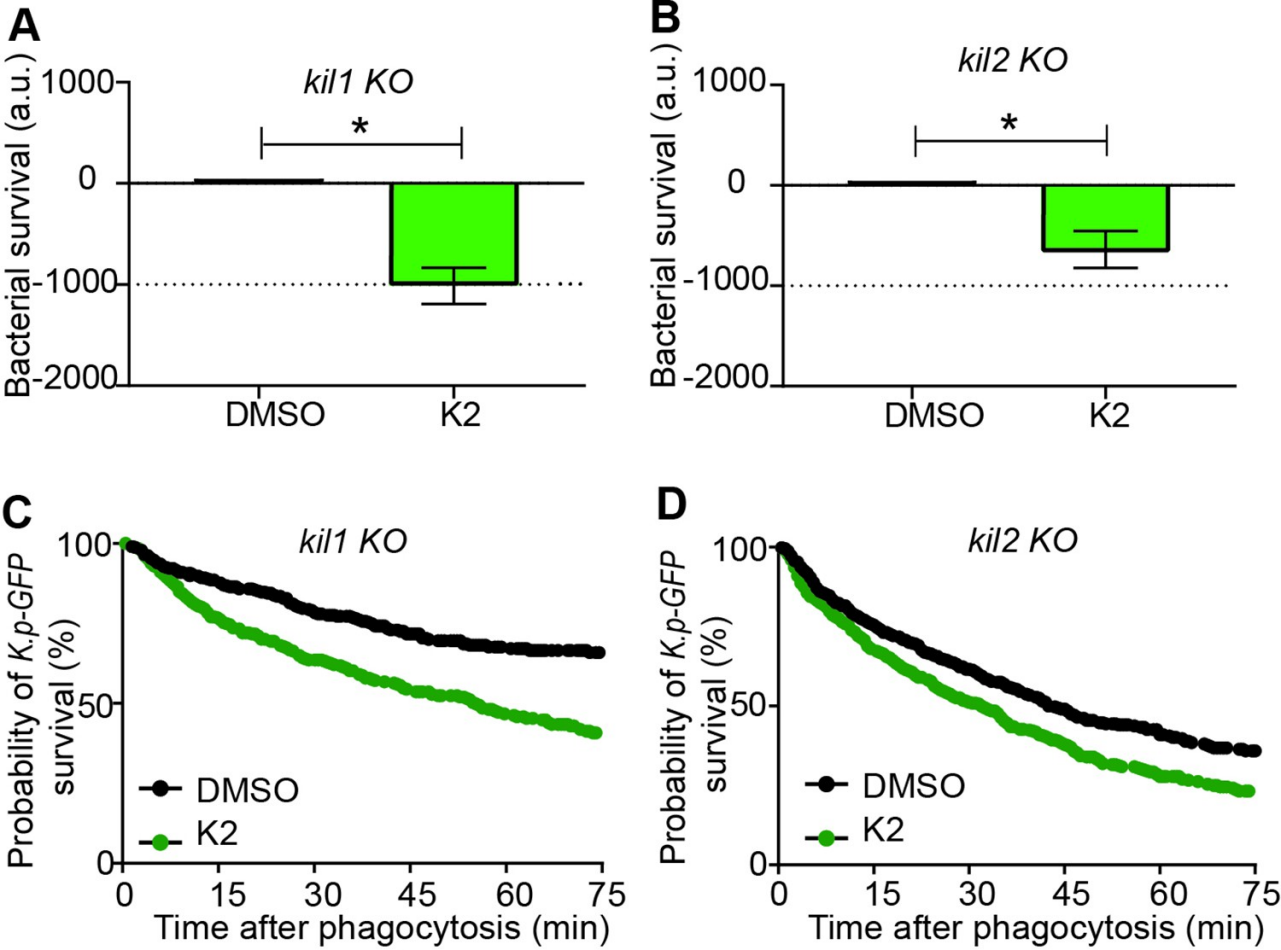
K2 increases intracellular killing of *K*. *pneumoniae* by *kil1* KO and *kil2* KO cells. The effect of the K2 compound on *K*. *pneumoniae* intracellular killing was assessed after ingestion by *kil1* KO cells (**A**) or by *kil2* KO cells (**B**). K2 (green) increased significantly bacterial killing in both mutant cells (mean ± SEM; * p<0.05 Mann-whitney test, *kil1* KO: N = 9; *kil2* KO: N = 13 independent experiments). **C, D**. The corresponding survival curves of ingested *K*. *pneumoniae* in *kil1* KO (C) and *kil2* KO cells (D) are shown.

We also tested whether K2 facilitates intracellular killing of *K*. *pneumoniae* in other *D*. *discoideum* killing-deficient mutants. Indeed, K2 facilitated killing of *K*. *pneumoniae* in *kil1* KO cells (Fig 5A and 5B) and, to a lesser extent, in *kil2* KO cells (Fig 5C and 5D).

### *K*. *pneumoniae* exposed to K2 are easier to kill

**Fig 6:**
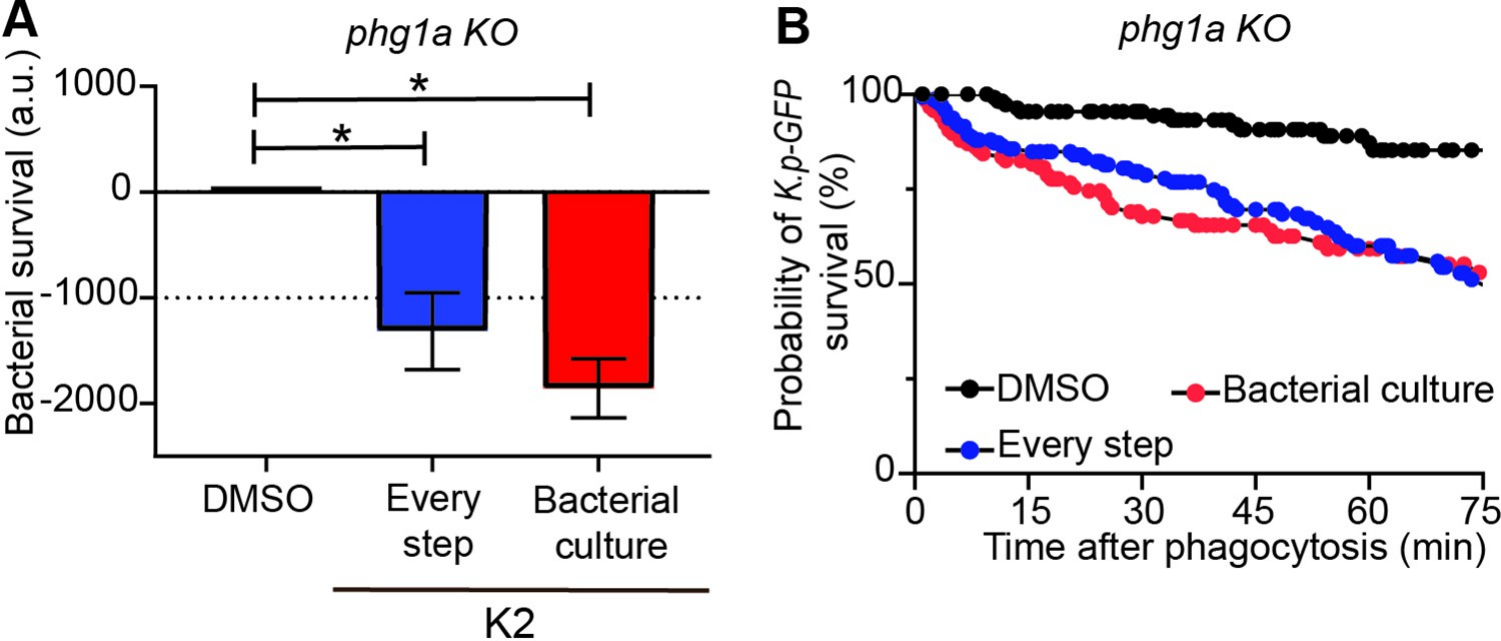
K2 treatment renders *K*. *pneumoniae* more susceptible to intracellular killing. **A.** The effect of K2 on intracellular killing was assessed by adding it at different steps of the experimental process. K2 was added either at every step (bacterial overnight culture, *D*. *discoideum* overnight culture and during the ingestion and killing of bacteria; blue), or only in the overnight bacterial culture (red), and compared to a treatment with DMSO (mean ± SEM; *: p<0.05; Kruskal-Wallis test, Dunn’s test. N = 5 independent experiments). **B.** The corresponding survival curves with K2 added at every step (blue) or only during the bacterial preculture (red) are shown.

In all experiments up to this point, the compounds were added 16 hours before the experiment both to the *K*. *pneumoniae* culture, and to the *D*. *discoideum* culture. The compounds were also present when cells and bacteria were mixed and phagocytosis and killing recorded. K2 could in principle act either on bacteria by fragilizing *K*. *pneumoniae*, or on *D*. *discoideum* cells by stimulating intracellular killing mechanisms. To distinguish between these two possibilities, we measured intracellular killing of bacteria that had been grown in the presence of K2, but omitting K2 in the *D*. *discoideum* preculture and during the ingestion and killing of bacteria. In this setting, bacteria were washed twice before use and *D*. *discoideum* cells were not exposed to the K2 compound. Exposing bacteria to K2 during their growth was sufficient to facilitate their killing by *D*. *discoideum* cells (Fig 6). This result suggests that K2 acts primarily on *K*. *pneumoniae*, making it more susceptible to intracellular killing in *D*. *discoideum* phagosomes.

**Fig 7:**
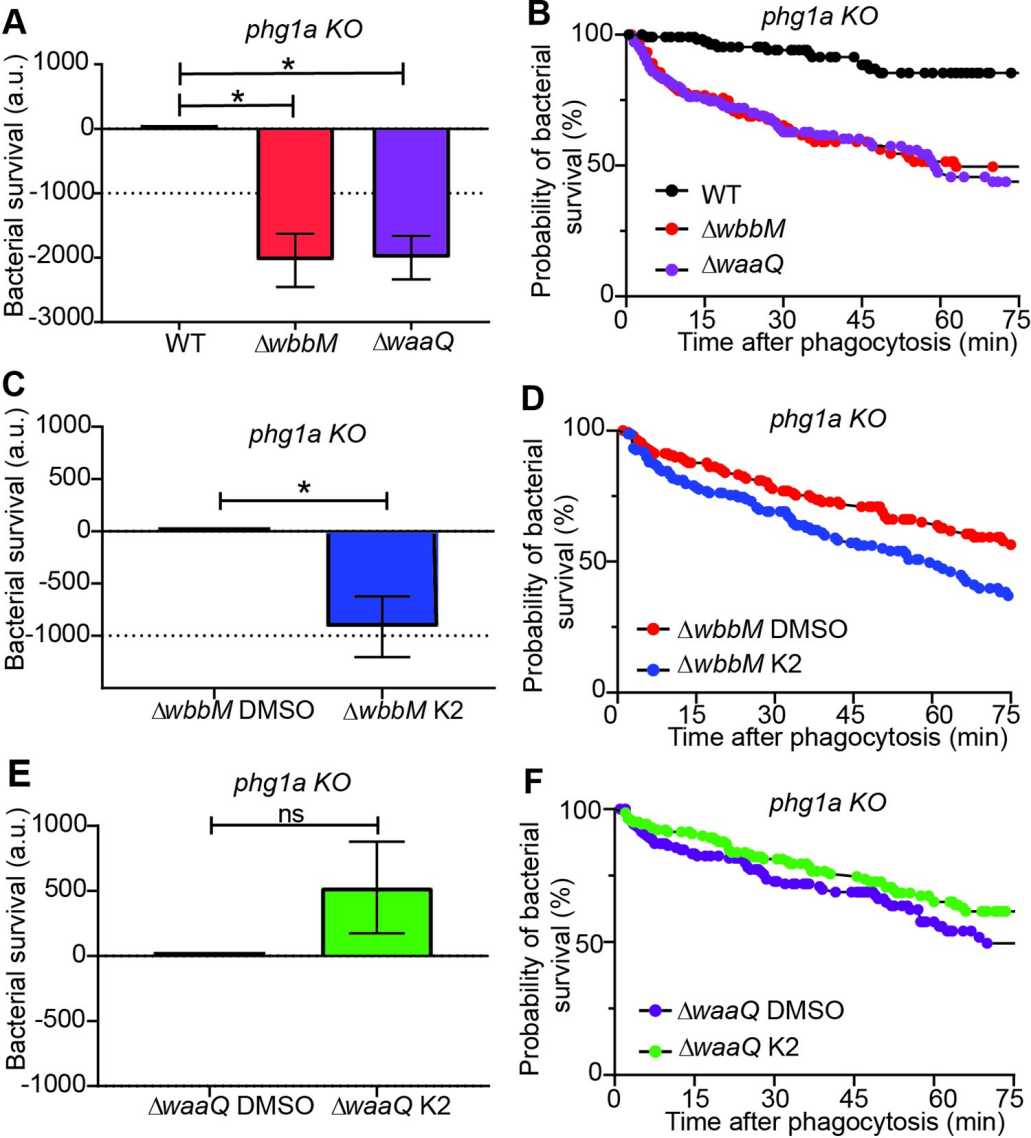
Intracellular killing of *K*. *pneumoniae* mutants. **A.** Intracellular killing of *K*. *pneumoniae (*WT: black, Δ*wbbM*: red, Δ*waaQ*: purple) in *phg1A* KO *D*. *discoideum* was assessed as described in the legend to Fig 3 (mean ± SEM; *: p < 0.05; Mann-whitney test. Δ*wbbM*: N = 4; Δ*waaQ*; N = 5 independent experiments) **B.** The corresponding survival curves of Δ*wbbM* (red), Δ*waaQ* (purple) and WT *K*. *pneumoniae* (black) are shown. **C.** Exposure to K2 further increased the intracellular killing of Δ*wbbM K*.*pneumoniae* in *phg1a* KO cells (mean ± SEM; *: p < 0.05; Mann-whitney test. N = 5 independent experiments). **D**. The corresponding survival curves of Δ*wbbM K*. *pneumoniae* treated with DMSO (red) or K2 (blue) are shown. **E.** Exposure to K2 did not further increase intracellular killing of Δ*waaQ K*. *pneumoniae* in *phg1a* KO cells (mean ± SEM; Mann-whitney test. N = 5 independent experiments). **F.** The corresponding survival curves of Δ*waaQ K*. *pneumoniae* treated with DMSO (purple) or K2 (green) are shown.

In order to better delineate the mode of action of the K2 compound, we tested its effect on *K*. *pneumoniae* mutants with altered LPS synthesis. The *wbbM* gene is necessary for O-antigen coupling to bacterial LPS, while *waaQ* participates in the inner core biosynthesis. Genetic inactivation of *wbbM* prevents the addition of O-antigens to the LPS core. Genetic inactivation of *waaQ* results in loss of the heptose III side branch of the LPS core, a structure critical for outer membrane stability [17]. Intracellular killing of Δ*waaQ* or Δ*wbbM K*. *pneumoniae* by *phg1A* KO *D*. *discoideum* was greatly facilitated compared to killing of WT *K*. *pneumoniae* (Fig 7A, 7B). K2 further stimulated killing of Δ*wbbM* bacteria (Fig 7C, 7D), but not that of Δ*waaQ* bacteria (Fig 7E, 7F). This observation indicates that when the LPS layer protecting the bacteria is altered by a Δ*waaQ* genetic inactivation, no additional effect of K2 on bacterial survival is visible. As detailed below, this suggests that K2 may act by perturbing the protective effect of the outer bacterial layer which includes LPS, but may also include other elements.

Intracellular killing of bacteria can be at least partly reproduced in vitro by exposing bacteria to extracts from *D*. *discoideum* cells and following bacterial lysis [20]. Extracts from *phg1A* KO cells lysed bacteria less rapidly than extracts from WT *D*. *discoideum* (S3 Fig in S1 File). When bacteria were grown in the presence of K2, bacterial lysis by *D*. *discoideum* extracts was accelerated (S3 Fig in S1 File). This result reinforces the notion that the main effect of K2 is to make *K*. *pneumoniae* bacteria more succeptible to attack by cellular antibacterial mechanisms.

### K2 treatment disrupts the protective effect of the LPS layer in *K*. *pneumoniae*

**Fig 8:**
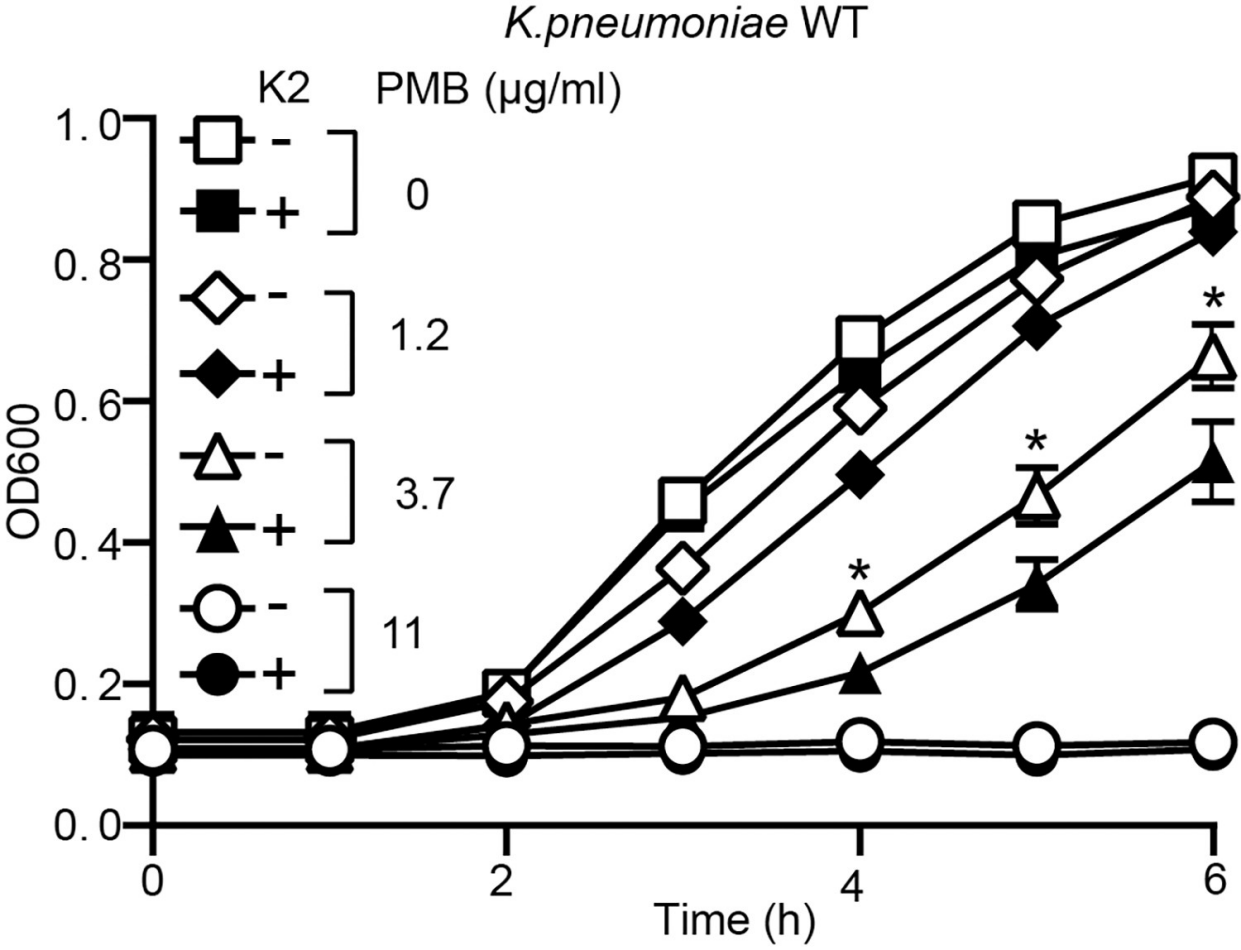
K2 increases the sensitivity of *K*. *pneumoniae* to polymyxin B. WT *K*. *pneumoniae* were grown overnight in the presence or absence of K2. The bacteria were then diluted and their growth was assessed for 6 h in the continued presence or absence of K2 and in the presence of increasing concentrations of polymyxin B (PMB: 0-11 μg/ml). While exposure to K2 did not alter the growth of *K*. *pneumoniae*, it increased its sensitivity to polymyxin B as evidenced by a slower growth in the presence of 1.2 or 3.7 μg/ml of PMB (mean ± SEM; *: p < 0.05; Mann-whitney test. PMB 3.7 μg/ml: N = 12 independent experiments).

Polymyxin B kills bacteria by disrupting their membranes [21, 22]. To be effective polymyxins need first to cross the protective layer formed by bacterial LPS. Consequently, polymyxins inhibit more efficiently growth of bacteria exhibiting a disorganized LPS layer. A standard bacterial growth inhibition assay failed to reveal any synergistic effect between K2 and polymyxin B or other antibiotics (S4 Fig in S1 File), however this assay may fail to detect subtle effects of K2 on bacterial growth kinetics. We thus measured growth of *K*. *pneumoniae* in LB containing increasing concentrations of polymyxin B (Fig 8). In the absence of K2, a concentration of 11 μg/ml of polymyxin B fully inhibited growth of *K*. *pneumoniae*, and polymyxin B at 3.7 μg/ml and 1.2 μg/ml partially inhibited bacterial growth. K2 alone (30μM) failed to inhibit bacterial growth. However, when both polymyxin B (3.7 μg/ml) and K2 were added to bacteria, the bacterial growth was slower than when only polymyxin B was used (Fig 8). When a similar experiment was performed with tetracyclin instead of polymyxin B, K2 did not potentiate the effect of tetracycline on bacterial growth (S5 Fig in S1 File). These results suggest that exposure to K2 disorganizes the LPS layer of *K*. *pneumoniae* bacteria, making them more sensitive to polymyxin B.

**Fig 9:**
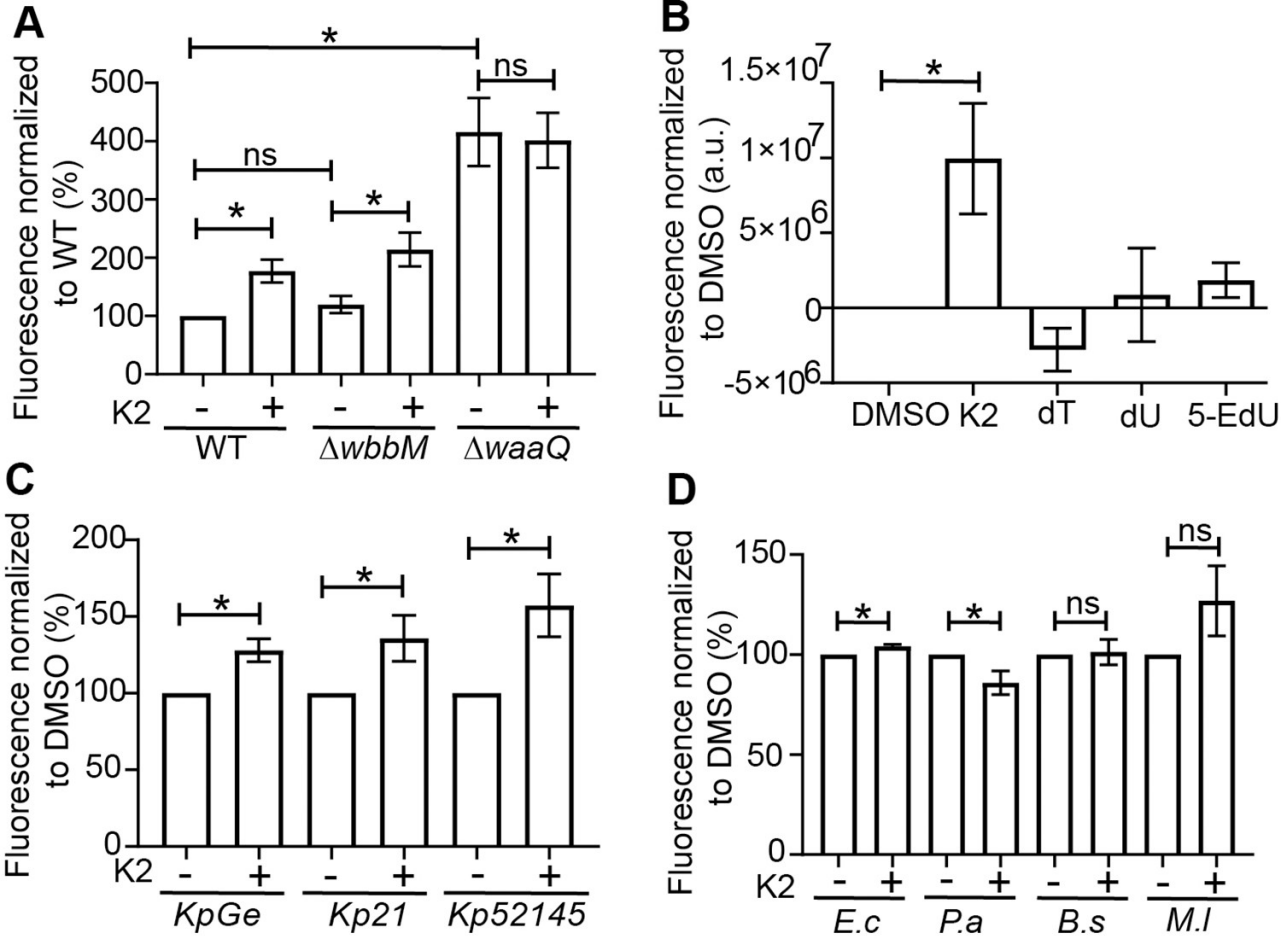
K2 treatment increases access of a hydrophobic probe to the bacterial membrane of *K*. *pneumoniae*. **A.** In order to assess the efficacy with which the LPS shielded the bacterial membrane, bacteria were exposed to the fluorescent probe 1-N-phenylnaphthylamine (NPN), and fluorescence was recorded, providing a measure of the insertion of NPN in the bacterial membrane. The membrane of Δ*waaQ* mutant *K*. *pneumoniae* was more accessible to NPN than that of WT and Δ*wbbM* mutant. In K2-treated bacteria, access of NPN increased in WT and in Δ*wbbM* but not in Δ*waaQ* bacteria (mean ± SEM; *: p<0.05; Kruskal-Wallis test; N = 5 independent experiments). **B.** Three chemical analogs of K2 (dT = deoxythymidine, dU = deoxyuridine and 5-EdU = 5-Ethynyl-2’-deoxyuridine) were tested for their ability to increase the membrane accessibility of NPN. K2 was the only compound that increased significantly the accessibility of the outer membrane of *K*. *pneumoniae* to NPN (mean ± SEM; *: p<0.05; Kruskal-Wallis test; DMSO, K2: N = 6; and N = 5 independent experiments for the three analogs of K2). **C, D.** The effect of K2 was tested on three different strains of *K*. *pneumoniae* (KpGE, Kp21 and Kp52145) as well as *Escherichia coli (E*.*c)*, *Pseudomonas aeruginosa (P*.*a)*, *Bacillus subtilis (B*.*s)* and *Microccocus luteus (M*.*l)*. K2 increased NPN incorporation in *K*. *pneumoniae*, but exhibited little or no effect on other bacteria. (mean ± SEM; *: p<0.05; Mann-whitney test. N = 8 independent experiments).

In order to evaluate directly the protective effect of the LPS layer in different strains and conditions, we measured the accessibility of the bacterial membrane using the hydrophobic fluorescent probe 1-N-phenylnaphthylamine (NPN). When NPN inserts in the outer lipidic membrane of bacteria, its fluorescence strongly increases [23]. In WT *K*. *pneumoniae*, the LPS layer limits the accessibility of the outer membrane and largely excludes NPN (Fig 9A). Genetic inactivation of *waaQ* significantly increased access of NPN to the outer membrane, while genetic inactivation of *wbbM* did not (Fig 9A). K2 increased outer membrane accessibility in WT and Δ*wbbM*, but not in Δ*waaQ K*. *pneumoniae* (Fig 9A). This effect was seen at concentrations of K2 (3μM and above) similar to those required to render *K*. *pneumoniae* easier to kill (S6A Fig in S1 File), and was detectable after 8 h of growth of bacteria in the presence of K2 (S6B Fig in S1 File). Three close chemical analogs of K2 were also tested in this assay (S2 Fig in S1 File) and did not modify outer membrane accessibility (9b Fig), indicating that the action of K2 is highly specific. An increased accumulation of NPN can in principle be caused by an increase in bacteria outer membrane accessibility, or by an inhibition of multi-drug resistance pumps [24]. Indeed outer membrane accessibility was increased by CCCP, an inhibitor of multi-drug resistance pumps, but the effects of CCCP and NPN were additive (S7 Fig in S1 File), suggesting that NPN does not act by inhibiting multi-drug resistance pumps.

In order to characterize the spectrum of action of K2, we also determined whether it affected outer membrane accessibility in other strains of bacteria. In addition to the non-virulent KpGE strain of *K*. *pneumoniae* used in this study, we tested two virulent strains of *K*. *pneumoniae* (Kp21and Kp52145), as well as two other Gram-negative (*Escherichia coli*, *Pseudomonas aeruginosa*) and two Gram-positive (*Micrococcus luteus*, *Bacillus subtilis*) bacterial strains. K2 increased the accessibility of the outer membrane for the three *K*. *pneumoniae* strains tested (Fig 9C), but did not show a significant effect on the other bacteria tested (Fig 9D).

**Fig 10:**
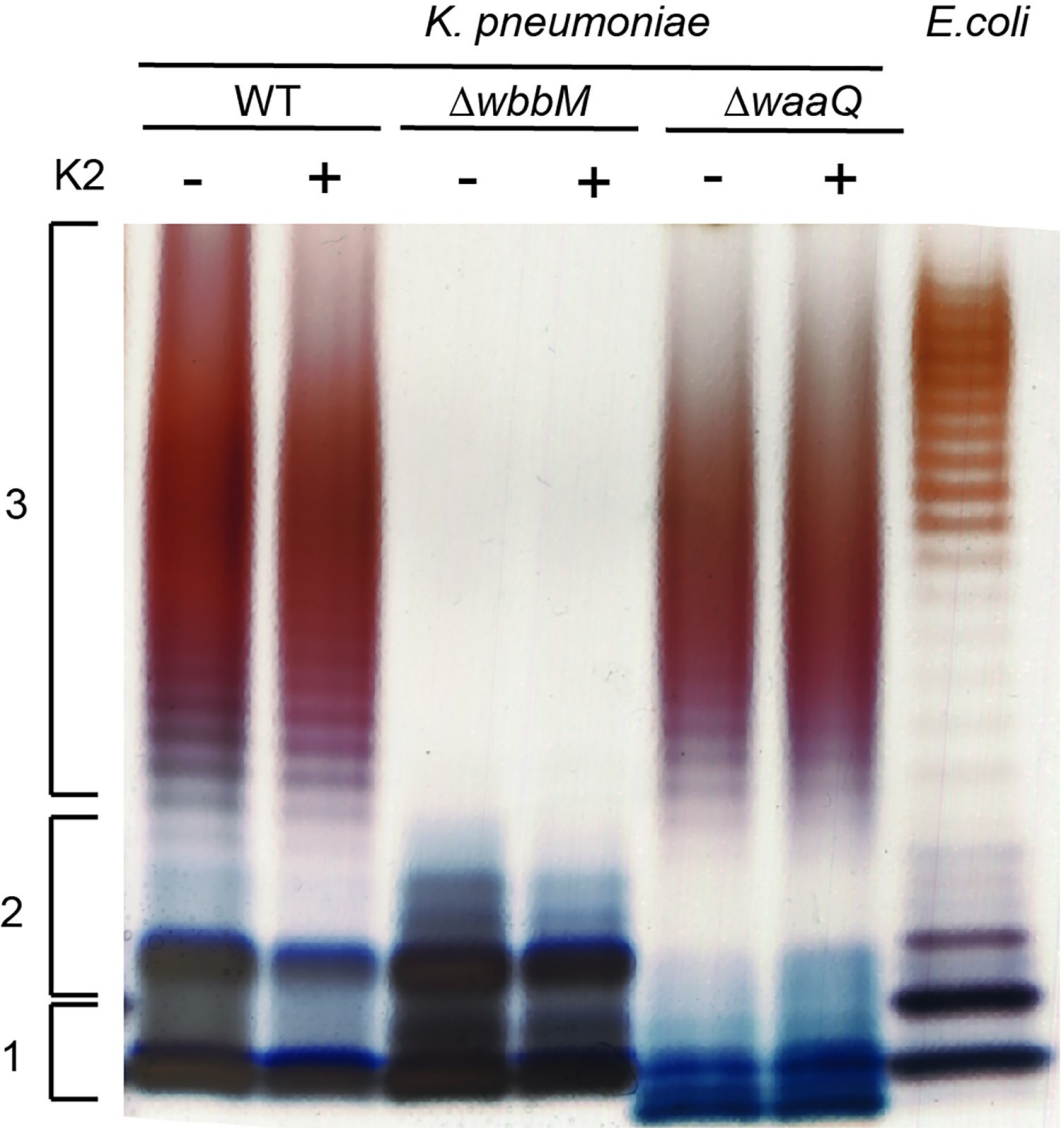
K2 treatment does not visibly affect LPS structure. Bacteria were grown overnight in the presence or absence of K2. LPS from *K*. *pneumoniae* WT and mutant strains (Δ*wbbM*, Δ*waaQ*) were then purified and analyzed by SDS-PAGE electrophoresis and silver staining. LPS extracted from *E*. *coli* (serotype O111:B4) was used for comparison. The LPS of WT *K*. *pneumoniae* showed the three main forms of the LPS: lipid A (1), lipid A+ oligosaccharide core (2), lipid A + core + O-antigen [27]. As expected, the LPS from Δ*wbbM* bacteria lacked the O-antigen, and Δ*waaQ* displayed a smaller oligosaccharide core that migrated further in the gel. No visible alteration of LPS structure was observed in K2-treated bacteria.

Finally, we assessed directly the effect of K2 on the structure of bacterial LPS. For this, we purified LPS from *K*. *pneumoniae* and analyzed its structure by SDS-polyacrylamide gel electrophoresis (Fig 10). LPS purified from WT *K*. *pneumoniae* exhibited a lipid A anchor, coupled to an oligosaccharide core, and to a repetitive glycan polymer forming the O-antigen. Incomplete structures composed of only lipid A, or lipid A coupled to the oligosaccharide core, were visible (Fig 10). As expected, LPS from Δ*wbbM K*. *pneumoniae* was devoid of the O-antigen, and LPS from Δ*waaQ* exhibited an incomplete (smaller) oligosaccharide core that migrated faster in the gel (Fig 10). Upon treatment of bacteria with K2, these LPS profiles remained unchanged (Fig 10). We also visualized the bacterial envelope by electron microscopy, and exposure to K2 did not visibly modify the structure of the bacterial envelope (S8 Fig in S1 File). These observations indicate that K2 does not affect the structure of the bacterial envelope in a manner detectable with these techniques.

## Discussion

In this study, we identified three compounds that modify the interaction between *K*. *pneumoniae* bacteria and phagocytic *D*. *discoideum* cells. We then characterized in more detail the mode of action of one of them, K2, which increases the intracellular killing of *K*. *pneumoniae*. There are in principle two ways by which intracellular killing can be facilitated: either by stimulating the intracellular killing mechanisms, or by rendering bacteria more susceptible to intracellular killing. K2 clearly falls into the second category, since bacteria grown in the presence of K2 are more easily killed when they are ingested by *D*. *discoideum*, as well as when they are exposed to *D*. *discoideum* extracts *in vitro*. In bacteria treated with K2, the outer membrane is less protected from outer agents as revealed by their increased sensitivity to polymyxin B and by the observation that their outer membrane is more accessible to a hydrophobic fluorescent probe. On these four counts (faster intracellular killing in vivo and in vitro, increased sensitivity to polymyxin B, increased accessibility of bacterial membrane) the phenotype of K2-treated bacteria is similar to that produced by genetic inactivation of *waaQ*, which alters the structure and function of the LPS layer. The structure of LPS was however not visibly altered in K2-treated bacteria. We conclude that K2 acts by rendering the bacterial membrane more accessible to antibacterial agents, but our results do not indicate that K2 alters the structure of LPS, and do not identify the primary target of the K2 compound. Many antibacterial agents bind to LPS and increase the accessibility of the outer membrane, notably cationic agents, ion chelators, or polymyxins [25, 26]. This is however unlikely to be the case for K2, since its effect only appeared after prolonged (8h) exposure of bacteria to K2. K2 is an analog of thymidine. It may affect genome replication or gene expression and indirectly impact the organization of the bacterial envelope. Further studies will be necessary to determine by which molecular mechanism K2 modifies the properties of bacterial envelope.

In principle a compound affecting the resistance of bacteria to intracellular killing could limit the ability of *K*. *pneumoniae* to create virulent infections in patients. In this respect, it is worth noting that Δ*waaQ* and Δ*wbbM* mutant *K*. *pneumoniae* strains completely lost the ability to mount a lung infection in mice [11], although their intracellular killing was only partially restored in *phg1A* KO *D*. *discoideum* cells. This observation suggests that even a small increase in the sensitivity of *K*. *pneumoniae* bacteria to intracellular killing can strongly diminish their capacity to infect patients.

K2 is 5-ethyl-2’-deoxyuridine, also known as edoxudine. It was used until 1998 as a topical antiviral drug, to treat genital herpes simplex infections [27] and its properties have been carefully studied. It inhibits viral replication in vitro at concentrations ranging from 5 to 50 μM [28]. When administered orally or intravenously, concentrations readily reach values higher than 10 μM in the blood as well as in the lungs [29, 30]. Since K2 decreases *K*. *pneumoniae* virulence at a concentration of 3 μM, we speculate that it could be used to treat *K*. *pneumoniae* infections. This would be particularly useful when faced with bacteria resistant to multiple antibiotics against which therapeutic options are currently very limited. *K*. *pneumoniae* infections can cause severe necrotizing skin infections, often resistant to classical antibiotics, and sometimes fatal [31]. Because K2 was initially approved as a topical drug, it may be used in such situations, possibly as a complement to classical antibiotic treatments. Repurposing of approved drugs to treat bacterial infections is an interesting strategy because information is already available about pharmacological and toxicological profiles in preclinical and clinical studies [32]. In this perspective it is encouraging to find that K2 increases the outer membrane accessibility of three different strains of *K*. *pneumoniae*, including two well-characterized pathogenic strains. Detailed studies will however be necessary to determine if K2 acts on pathogenic *K*. *pneumoniae* strains in infected patients.

### Experimental procedures

#### Cells and reagents

All *D*. *discoideum* cells used in this study were derived from the parental DH1-10 strain [33], referred to as wild-type (WT). *Phg1a* KO, *kil1* KO and *kil2* KO cells were described previously [11, 16]. *D*. *discoideum* cells were grown in HL5 medium [34] at 21°C. When indicated, *D*. *discoideum* cells were grown on a lawn of *K*. *pneumoniae*, as previously detailed [34].

The parental *K*. *pneumoniae* strain used in this study is the sequenced non-pathogenic laboratory strain KpGE strain [15]. It was grown in LB (lysogeny broth) at 37°C. Δ*waaQ* and Δ*wbbM* mutants of KpGE were described previously [11]. We used for screening a previously described collection of 1,099 compounds [35]. This collection comprises the 1,040 bioactive compounds of the NINDS custom collection 2 (Microsource Inc., Gaylordsville, CT), completed with 59 locally selected compounds. Three quarters of the compounds in the collection are FDA-approved. The three compounds selected (K1: CAS 138-14-7; K2: CAS 15176-29-1; K3: CAS 58-22-0) were re-ordered from Merck (Darmstadt, Germany).

#### Screening for inhibitors of bacterial virulence

Inhibitors of bacterial virulence were tested as previously described [34]. Briefly, in a 24-well plate containing 2 ml Standard Medium-agar (for 1 L: 10 g bacteriological peptone, 1 g bacto yeast extract, 2.2 g KH_2_PO_4_, 1 g K_2_HPO_4_, 1 g MgSO_4_:7H_2_O, 20% glucose), a 20 μl droplet of each compound was deposited in each well to a final concentration of 30 μM. KpGE bacteria (50 μl of overnight culture) were then added in each well and allowed to dry in a sterile cell culture hood for 2 h. *D*. *discoideum phg1a* KO cells (30,000, 10,000, 3,000, or 1,000 cells) were applied onto the bacterial lawn. After ten days, the plates were scanned with an Epson Perfection V850 Pro scanner. *phg1a* KO cells created phagocytic plaques (white) on the bacterial lawn (black) only when a compound facilitated their growth (Fig 1A). The growth of *phg1a* KO cells was scored from 4 (efficient growth of 1,000 cells) to 0 (no growth of 30,000 cells).

#### Antibiotic activity of compounds

An overnight bacterial culture of *K*. *pneumoniae* (300 μl) was spread on a Petri dish containing LB-agar. Paper discs with 20 μl of a 10 mM DMSO stock solution of each compound were then placed on the plates and the bacteria allowed to grow at 25°C overnight. Inhibition of bacterial growth around the disc reveals the antibiotic activity of compounds. DMSO was used as a negative control and tetracyclin (12 mg/ml) as a positive control.

To detect a putative additive effect of compounds when combined with antibiotics, bacteria were grown overnight with or without 30 μM of K2. *K*. *pneumoniae* bacteria (500 μl culture, 10^9^ cfu / mL) were then spread on a Petri dish containing LB-agar (with or without 30 μM of K2 added in the LB-Agar). Antibiotic discs were then deposited on the plates and bacteria were allowed to grow at 37°C overnight. Inhibition of bacterial growth around the disc revealed antibiotic activities.

To detect synergistic effect of polymyxin B and K2 on bacterial growth, bacteria were grown 16 h at 37°C in a shaken suspension of 2 ml LB containing 6 μl of DMSO with or without K2 (final concentration 30 μM). To test if K2 increases the susceptibility of *K*. *pneumoniae* to the antibiotic activity of polymyxin B or tetracycline, 50 μl of DMSO/K2-treated bacteria were transferred to 2 ml of LB supplemented with DMSO or K2. A range of concentrations of polymyxin B or tetracycline was also added to the culture. The cultures were grown at 37°C in a shaken suspension. An aliquot of 150 μl was taken every hour for 6 hours and placed in a 96-well plate (cell culture microplate, PS, F-Bottom, black, Greiner bio-one). The bacterial growth was determined by measuring the OD_600nm_ with a plate reader (SpectraMax Paradigm from Molecular Devices, SoftMaxPro 7.0.)

#### Intracellular killing of bacteria

To measure intracellular killing of ingested bacteria, GFP-expressing *K*. *pneumoniae* were grown 16 h at 37°C, as previsouly described [19]. The culture was washed twice in phosphate buffer (PB)-sorbitol (2 mM Na_2_HPO_4_, 14.7 mM KH_2_PO_4_, 100 mM sorbitol, pH 6.0) then resuspended in 1 ml PBS. The bacterial suspension was diluted 200 times in PBS, and 150 μL were mixed with 230,000 *D*. *discoideum* cells in a final volume of 250 μL PB-sorbitol, and deposited on a glass slide (μ-slide 8 well glass bottom, Ibidi GmbH). When indicated, 30 μM of compound or DMSO (0.3%) was added to each well. The cells were allowed to settle for 10 min, then imaged every 30 sec for 2 h with a Nikon Eclipse Ti2. At each time point, one picture (phase contrast and GFP fluorescence) was taken in five successive focal planes (step size 3 μm) to image the whole cell volume. ImageJ was used to analyze movies. Bacterial fluorescence decreased abruptly (typically a >90% drop within 30 sec), and this event indicated the time when the bacteria was killed. For each bacteria analysed (30 per independent experiment) the time of phagocytosis and the time of killing were recorded. Survival of phagocytosed fluorescent bacteria was computed using the Kaplan–Meier estimator. Statistical analysis was done using GraphPad Prism (V8.1.0). For each condition, at least three independent experiments were performed.

#### Bacterial lysis in vitro

Bacterial lysis in vitro was assessed as previously described [20]. Briefly, *D*. *discoideum* cells were washed twice in phosphate buffer (PB: 2 mM of Na2HPO4 and 14.7 mM of KH2PO4, pH 2.0) and lysed in 800 μL of Lysis buffer (50 mM of sodium phosphate buffer, pH2, 0.5% Triton X‐100) containing protease inhibitors (20 μg/mL of leupeptin, 10 μg/mL of aprotinin, 18 μg/mL of phenylmethylsulfonyl fluoride (PMSF) and 1.8 mg/mL of iodoacetamide (IAA)). The suspension was centrifuged (30,000 g for 60 min at 4°C), and the supernatant was collected and serially in Lysis buffer. Bacteriolytic activity was assessed by mixing in a microtiter plate 100 μL of cell extract with 100 μL of an overnight bacterial culture (grown in the presence or absence of 30 μM K2) washed once in sodium phosphate buffer (50 mM sodium phosphate buffer, pH2) and resuspended in the same buffer to a final optical density (450 nm) of 0.5. The decrease in turbidity (optical density at 450 nm) for 40 min at 21°C was measured with a plate reader (SpectraMax Paradigm from Molecular Devices, SoftMaxPro 7.0.).

#### Bacterial outer membrane accessibility

The fluorescent probe 1-*N*-phenylnaphthylamine (NPN; Sigma-Aldrich) was used to assess the accessibility of the outer membrane of bacteria as described previously [23]. The stock solution of NPN was prepared in acetone (0.5 mM), kept at room temperature and used within a week. The stock solution was diluted in HEPES buffer (5 mM, pH 7.2), to a concentration of 40 μM. Bacteria were grown overnight at 37°C in 2 ml LB with or without 30 μM of K2 and reached an OD_600nm_ of 5. The culture was diluted in 9 volumes of HEPES buffer, then 1ml was centrifuged 10 min at 10,000 rpm. Finally, the bacterial pellet was resuspended in 300 μl HEPES buffer and used as described below. For a standard experiment, a 96-well plate (cell culture microplate, PS, F-Bottom, black, Greiner bio-one) was filled with (a) HEPES buffer (200 μl), (b) HEPES buffer (150 μl) + NPN 40 μM (50 μl), (c) HEPES buffer (100 μl) + DMSO/K2-treated bacterial suspension (100 μl), or (d) HEPES buffer (50 μl) + NPN 40 μM (50 μl) + DMSO/K2-treated bacterial suspension (100 μl). The bacterial suspensions were added last, just before measuring fluorescence (360nm excitation, 405nm emission) with a microplate reader. For each well, the flurorescence (arbitrary units; a.u.) was divided by the OD_600nm_ to obtain the corrected fluorescence value.

To assess when the effect of K2 on the bacterial outer membrane accessibility appeared, bacteria were grown 16 h at 37°C in a shaken suspension of 3 ml LB. Bacteria (500 μl) were transferred in LB (20 ml supplemented with 60 μl DMSO or K2). The cultures were grown at 37°C in a shaken suspension for 24 h and aliquots were taken at the indicated times. Each aliquot was transferred in a well (OD_600nm_ of 0.2) and NPN incorporation measured as described above.

The efflux pump inhibitor carbonyl cyanide 3-chlorophenylhydrazone (CCCP) was obtained from Sigma-Aldrich. The experimental procedure to measure the accessibility of the outer membrane was slightly adapted: bacteria resuspended in 300 μl HEPES were treated (or not) with 100 μM of CCCP for 10 min at room temperature, centrifuged 10 min at 10,000 rpm and the pellet resuspended in 300 μl HEPES. The suspension was then used as described above in a 96-well plate to measure NPN incorporation.

### LPS purification

*K*. *pneumoniae* (KpGE) bacteria (WT, Δ*wbbM*, *ΔwaaQ*) were grown for 16 h at 37°C in a shaken suspension of 2 ml LB. As indicated 6 μl of DMSO or K2 (final concentration 30 μM) was added. The Intron Biotechnology kit was used to extract the LPS following manufacturer’s instructions. Briefly, a first lysis step with an organic solution containing phenol was used to disrupt the phospholipid and protein components of the cell membrane. Chloroform was then applied afterward to isolate RNA and genomic DNA/protein. LPS were precipitated at a high salt concentration, washed with 70% EtOH, and resuspended in 10mM Tris-HCl buffer (pH 8.0). Finally, LPS were separated in a 4-15% Mini-PROTEAN® TGX™ Precast protein gel from BioRad then silver-stained. First, the gel was fixed with a fixation solution (40% ethanol, 10% acetic acid, 50% water) for 30 minutes. Then, the gel was incubated overnight with a solution containing 30% ethanol, 260 μl glutaraldehyde (50%), 20% incubation solution (sodium acetate trihydrate 34% and sodium thiosulfate pentahydrate 1%) and 50% water. After the overnight incubation step, the gel was washed three times 5 min in water and exposed 40 min to the silver nitrate solution with 92 μl formaldehyde (36%). Finally, the development solution was applied (sodium carbonate solution and 10 μl formaldehyde 36%) until the appearance of the LPS profiles. A stop solution containing disodium EDTA dehydrate was added 15 s later.

#### Electron microscopy

To study bacterial morphology by electron microscopy, bacteria were treated overnight with DMSO or 30 μM K2 and then were fixed in 2% glutaraldehyde (1 h at room temperature) and post-fixed in 2% osmium tetroxide (1 h at 4°C), dehydrated and embedded in Epon resin and processed for conventional electron microscopy as previously described [36].

### Data availability

All data is available within the manuscript. Unprocessed experimental data is available online on a dedicated server at the following address: https://doi.org/10.26037/yareta:oqmmzyw6crhqtmxfsjhoe65cwa.

## Supporting information

S1 Data (ZIP)

S1 File (DOCX)

## Abbreviations

PMB: Polymyxin B
K2: 5-ethyl-2’-deoxyuridine = Edoxudine
5-EdU: 5-ethynyl-2’-deoxyuridine
dT: deoxythymidine
dU: deoxyuridine
DMSO: Dimethyl sulfoxide
NPN: 1-N-phenylnaphthylamine
LPS: lipopolysaccharides
