# Supplementary material for: 5-ethyl-2’-deoxyuridine fragilizes Klebsiella pneumoniae outer wall and facilitates intracellular killing by phagocytic cells
Source: PLoS One. 2022 Oct 31;17(10):e0269093. doi: 10.1371/journal.pone.0269093 (PMC9621411; doi:10.1371/journal.pone.0269093)
Supplement: S1 Data — (ZIP) [file pone.0269093.s001.zip › Primary/Experimental/Figure 1/Figure 1BC images.pdf]

The unprocessed images of the 24-well plates shown in Figure 1B and C are presented in Figure S1_raw images
