# Supplementary material for: 5-ethyl-2’-deoxyuridine fragilizes Klebsiella pneumoniae outer wall and facilitates intracellular killing by phagocytic cells
Source: PLoS One. 2022 Oct 31;17(10):e0269093. doi: 10.1371/journal.pone.0269093 (PMC9621411; doi:10.1371/journal.pone.0269093)
Supplement: S1 Data — (ZIP) [file pone.0269093.s001.zip › Primary/Experimental/Figure 10/Figure 10 image.pdf]

The unprocessed image of the acrylamide gel shown in Figure 10 is presented in Figure S10_raw images
