# Supplementary figures and images for: 5-ethyl-2’-deoxyuridine fragilizes Klebsiella pneumoniae outer wall and facilitates intracellular killing by phagocytic cells
Source: PLoS One. 2022 Oct 31;17(10):e0269093. doi: 10.1371/journal.pone.0269093 (PMC9621411; doi:10.1371/journal.pone.0269093)

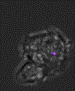

Supplement: S1 Data — (ZIP) [file pone.0269093.s001.zip › Primary/Experimental/Figure 3/Figure 3A/Images/Image8.png]

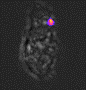

Supplement: S1 Data — (ZIP) [file pone.0269093.s001.zip › Primary/Experimental/Figure 3/Figure 3A/Images/image1b.png]

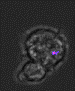

Supplement: S1 Data — (ZIP) [file pone.0269093.s001.zip › Primary/Experimental/Figure 3/Figure 3A/Images/Image7.png]

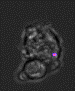

Supplement: S1 Data — (ZIP) [file pone.0269093.s001.zip › Primary/Experimental/Figure 3/Figure 3A/Images/Image6.png]

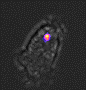

Supplement: S1 Data — (ZIP) [file pone.0269093.s001.zip › Primary/Experimental/Figure 3/Figure 3A/Images/Image4.png]

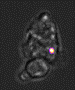

Supplement: S1 Data — (ZIP) [file pone.0269093.s001.zip › Primary/Experimental/Figure 3/Figure 3A/Images/Image5.png]

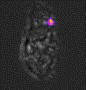

Supplement: S1 Data — (ZIP) [file pone.0269093.s001.zip › Primary/Experimental/Figure 3/Figure 3A/Images/Image1.png]

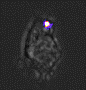

Supplement: S1 Data — (ZIP) [file pone.0269093.s001.zip › Primary/Experimental/Figure 3/Figure 3A/Images/Image2.png]

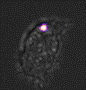

Supplement: S1 Data — (ZIP) [file pone.0269093.s001.zip › Primary/Experimental/Figure 3/Figure 3A/Images/Image3.png]
