# Supplementary material for: 5-ethyl-2’-deoxyuridine fragilizes Klebsiella pneumoniae outer wall and facilitates intracellular killing by phagocytic cells
Source: PLoS One. 2022 Oct 31;17(10):e0269093. doi: 10.1371/journal.pone.0269093 (PMC9621411; doi:10.1371/journal.pone.0269093)
Supplement: S1 File — (DOCX) [file pone.0269093.s002.docx]

**
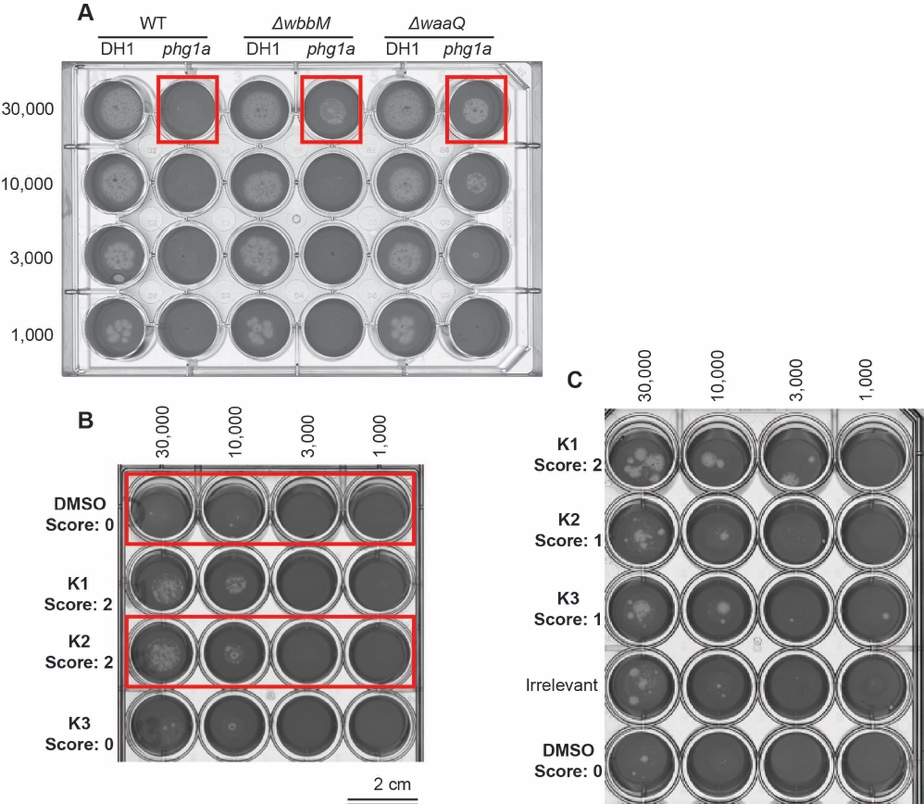
**

**Figure S1. Growth of *D. discoideum* cells on *K. pneumoniae* bacteria.** A. *Phg1A* KO cells did not grow on a lawn of WT *K. pneumoniae* bacteria, but they grew readily on bacterial mutants with decreased virulence (*ΔwaaQ, ΔwbbM*). Various numbers of *D. discoideum* cells (from 1,000 to 30,000) were deposited on a lawn of either WT or mutant (*ΔwaaQ, ΔwbbM*) *K. pneumoniae* bacteria. Wild-type (DH1) *D. discoideum* cells formed a phagocytic plaque (clear) in the bacterial lawn (dark) irrespective of the *K. pneumoniae* strain used. On the contrary, even 30,000 *phg1A* KO *D. discoideum* failed to clear WT *K. pneumoniae* bacteria. Growth of *phg1A* KO *D. discoideum* cells was partially restored on a lawn of mutant bacteria: phagocytic plaques were seen when 30,000 cells were deposited on *ΔwbbM K. pneumoniae,* and when 10,000 cells or more were deposited on *ΔwaaQ K. pneumoniae*. The three elements used in Figure 1B with enhanced contrast are boxed. B. K1, K2 and K3 compounds restore growth of *D. discoideum* cells on *K. pneumoniae* bacteria. As described above, various numbers of *phg1A* KO *D. discoideum* cells (from 1,000 to 30,000) were deposited on a lawn of WT *K. pneumoniae* in the presence of DMSO, or of the indicated compounds (K1, K2 or K3, 30μM). The growth score for each condition is indicated.The elements used in Figure 1C with enhanced contrast are boxed. Scale bar: 2 cm. C. An independent replicate of the experiment shown in B, shows similar, but slightly different results.

**
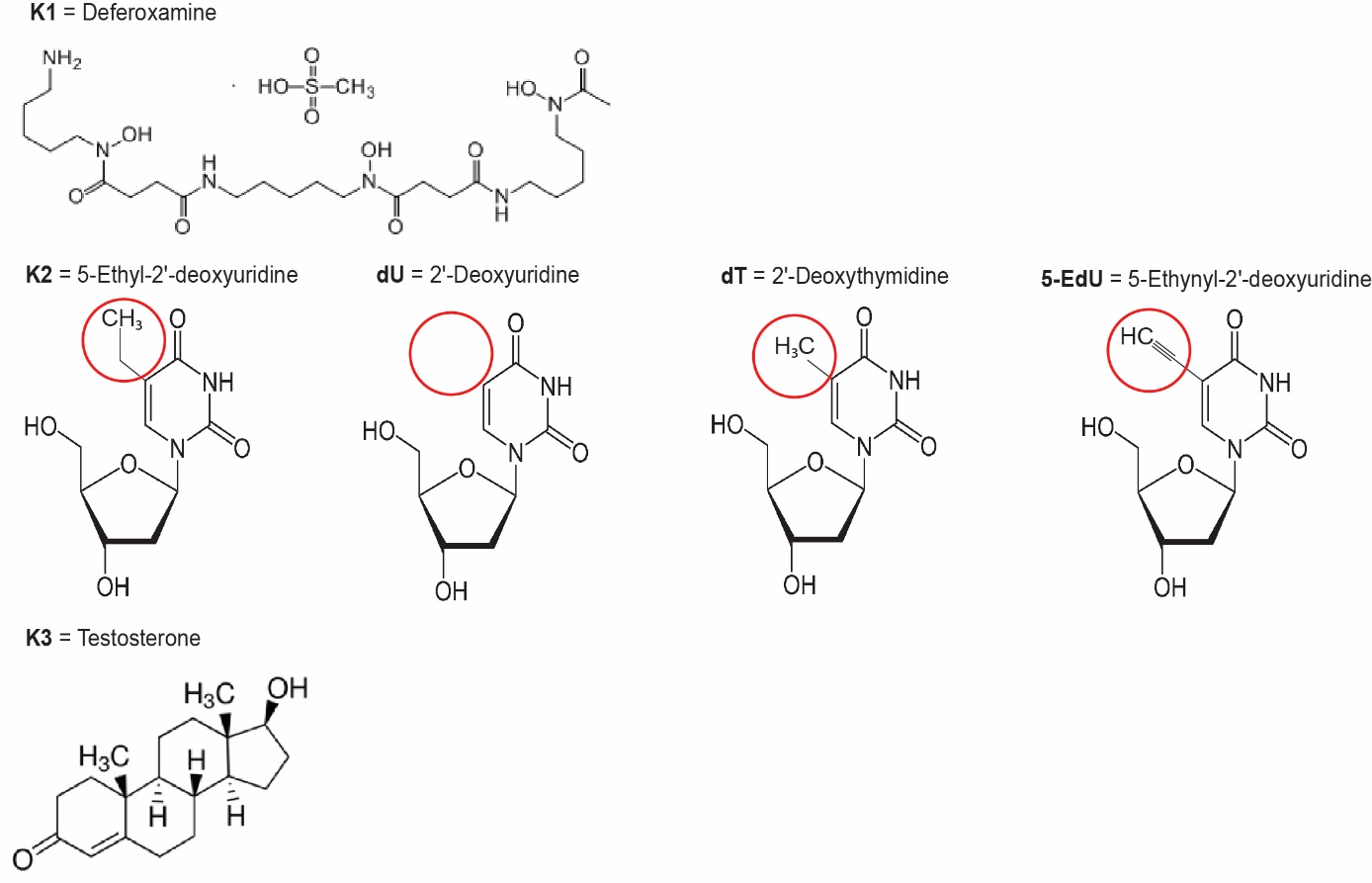
**

**Figure S2. Chemical structure of selected compounds.** Chemical structures of compounds K1, K2 and K3, and of three K2 analogs: dT=deoxythymidine, dU=deoxyuridine and 5-EdU=5-Ethynyl-2'-deoxyuridine. A red circle shows the site modified in K2 analogs.

**
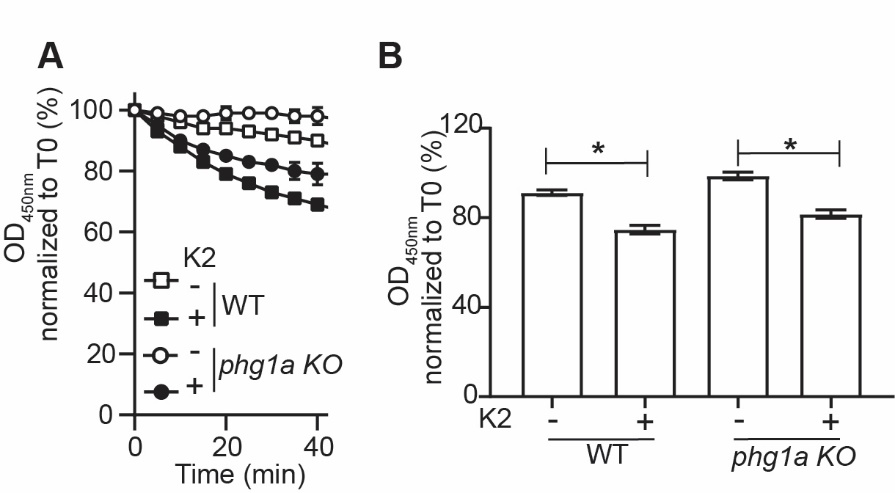
**

**Figure S3. K2 treatment increases the sensitivity of *K. pneumoniae* to the bacteriolytic activity of a *D. discoideum* extract.**

**A.** K2- or DMSO-treated bacteria were exposed to an extract of WT or *phg1a KO D. discoideum* cells and the OD_450nm_ was recorded over 40 min. The corresponding curves showing the lysis are shown. **B**. Bacterial lysis after 30 min of exposure is displayed (mean ± SEM; *: p<0.05; Wilcoxon t-test, WT N=15; *phg1a* KO N=8 independent experiments).

**
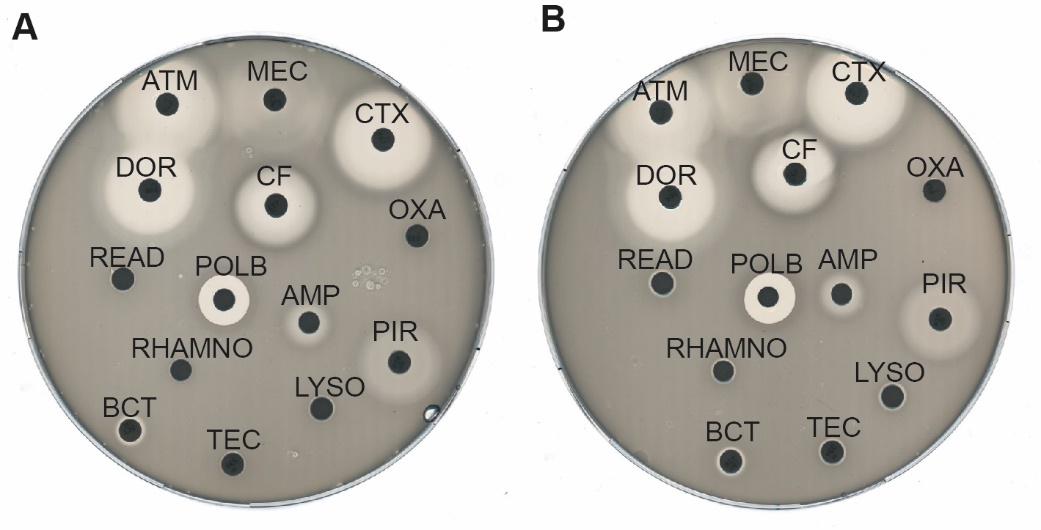
**

**Figure S4. K2 does not increase the bactericidal activity of antibiotics.** *K. pneumoniae* grown overnight with DMSO **(A)** or K2 **(B)** were spread on LB-agar plates containing K2 or DMSO. Antibiotic discs were then placed on the Agar and the plate incubated overnight at 37°C. None of the antibiotics tested displayed a larger halo of growth inhibition in the presence of K2 compared to the control (DMSO). CTX: cefotoxamine 30 µg; CF: cephalotin 30 µg; AMP: ampicillin 30 µg; PIR: piperacillin 100 µg; OXA: oxacillin 5 µg; MEC: mecillinam 10 µg; TEC: teicoplanin 30 µg; DOR: doripenem 10 µg; ATM: aztreonam 30 µg; BCT: bacitracine 130 µg; POLB: polymyxin B 50 µg; RHAM: Rhamnoplanin 50 µg; LYSO lysozyme 20 µg; READ Ready-Lyse 500U.

**
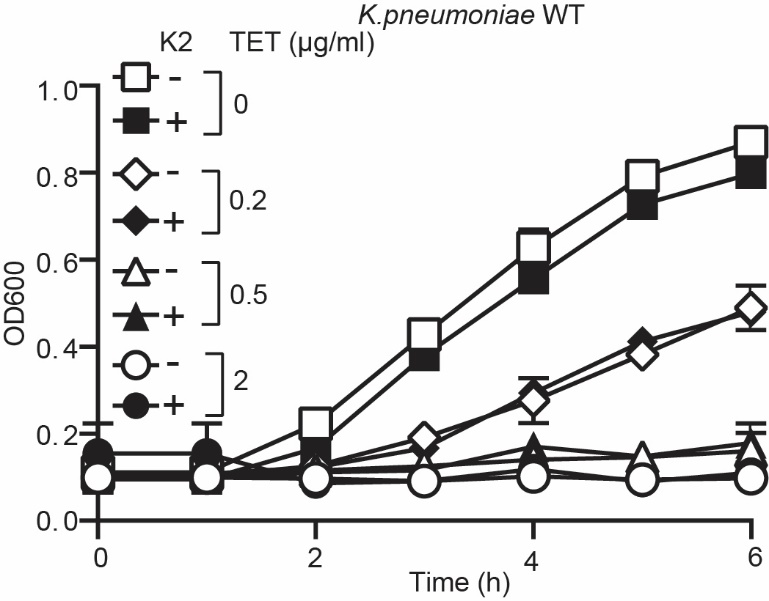
**

**Figure S5. K2 treatment does not increase the sensitivity of *K. pneumoniae* to tetracycline.** *K. pneumoniae* were grown overnight in the presence or absence of K2. The bacteria were then diluted and their growth was assessed for 6 h in the continued presence or absence of K2 and in the presence of increasing concentrations of tetracycline (TET: 0-2 µg/ml). K2-treated bacteria did not display an increased sensitivity to the antibiotic activity of tetracycline compared with the control (mean ± SEM; N=3-5 independent experiments).

**
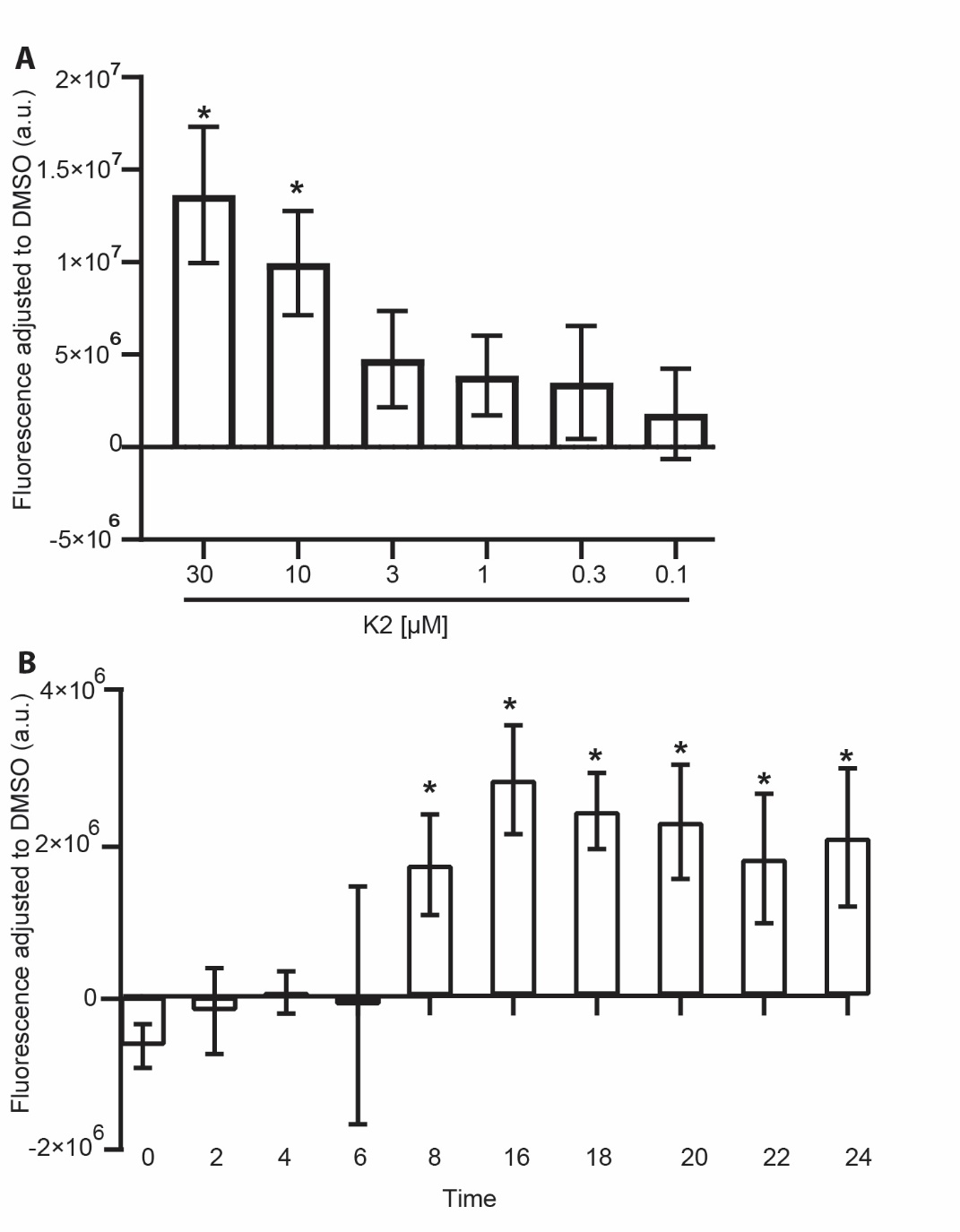
**

**Figure S6. K2 effect on NPN incorporation: required concentration and kinetics. A.** The effect of K2 on the accessibility of the outer membrane of *K. pneumoniae* to the fluorescent probe 1-N-phenylnaphthylamine (NPN) was determined after growing *K. pneumoniae* overnight in the presence of increasing concentrations of K2. K2 was effective at 10 µM and 30 µM. (mean ± SEM; *: p<0.05; Kruskal-Wallis test; N=6 independent experiments). **B.** In order to assess how rapidly the effect of K2 on the bacterial outer membrane accessibility appeared, *K. pneumoniae* bacteria were grown in the presence or absence of K2 (30µM), and the accessibility of the bacterial outer membrane was measured at the indicated times over 24 hours (mean ± SEM; *: p<0.05; Kruskal-Wallis test; N= 5-6 independent experiments).

**
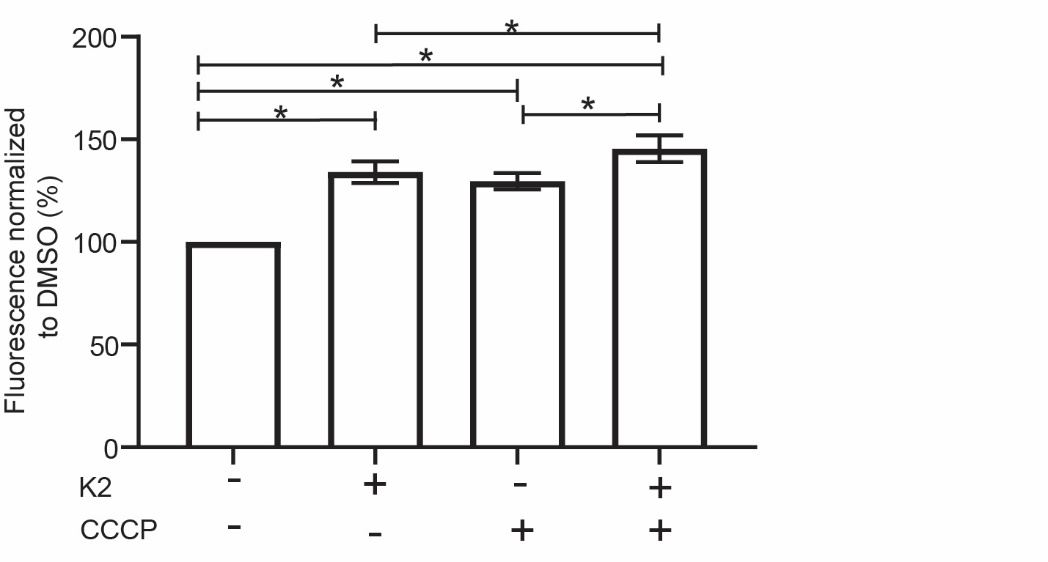
**

**Figure S7. K2 and the efflux pump inhibitor CCCP show additive effects on bacterial membrane accessibility.** Bacteria were treated with K2 or CCCP and the incorporation of NPN in their membrane assessed. A treatment with K2 or CCCP increased the accessibility of the bacterial outer membrane to NPN. K2 and CCCP showed additive effects on the accessibility of the outer membrane suggesting that the two compounds do not act on the same target (mean ± SEM; * p<0.05 Wilcoxon test; N=12 independent experiments).

**
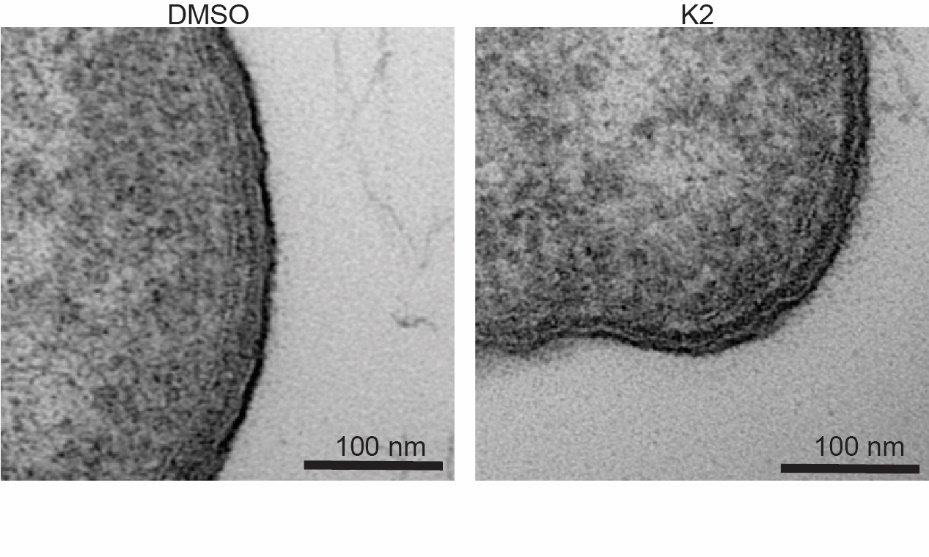
**

**Figure S8. K2 treatment does not visibly affect the ultrastructure of the *K. pneumoniae* envelope.**

Electron micrographs of K. pneumoniae bacteria grown in the presence of absence of K2. The two bacterial membranes are visible. The KpGE strain does not exhibit a capsule. K2-treated bacteria do not display an altered structure compared to control DMSO-treated bacteria (Scale bar: 100 nm).


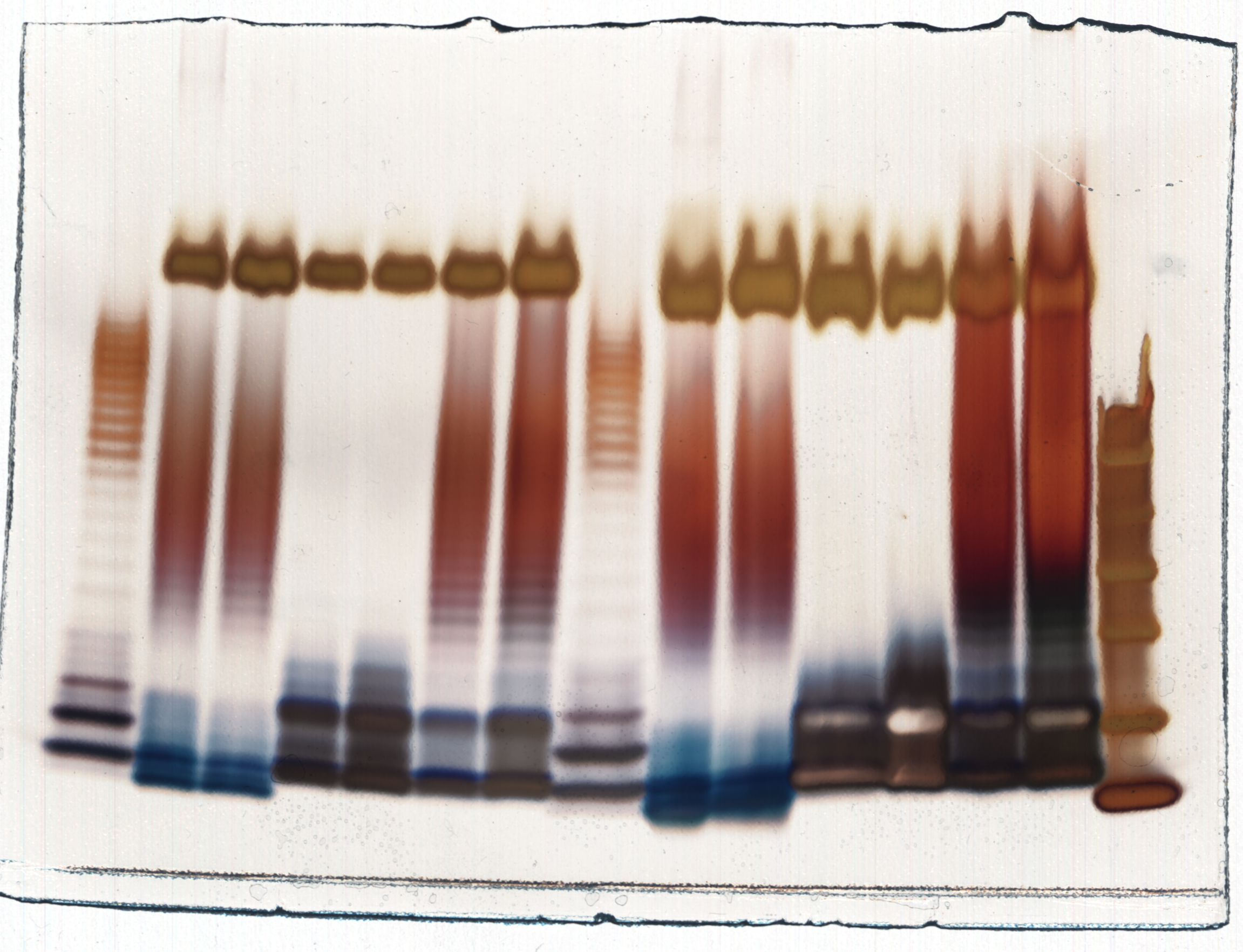


**Figure S9. K2 treatment does not visibly affect LPS structure.** This unprocessed image was used to construct Figure 10 in the accompanying manuscript.
